# Updates on drug designing approach through computational strategies: a review

**DOI:** 10.2144/fsoa-2022-0085

**Published:** 2023-05-04

**Authors:** Iqbal Azad, Tahmeena Khan, Naseem Ahmad, Abdul Rahman Khan, Yusuf Akhter

**Affiliations:** 1 Department of Chemistry, Integral University, Dasauli, P.O. Bas-ha, Kursi Road, Lucknow, 226026, UP, India; 2 Department of Biotechnology, Babasaheb Bhimrao Ambedkar University, Vidya Vihar, Raebareli Road, Lucknow, UP, 2260025, India

**Keywords:** computer-aided drug discovery, drug targets, medicinal, molecular docking, molecular dynamic, repurposing

## Abstract

The drug discovery and development (DDD) process in pursuit of novel drug candidates is a challenging procedure requiring lots of time and resources. Therefore, computer-aided drug design (CADD) methodologies are used extensively to promote proficiency in drug development in a systematic and time-effective manner. The point in reference is SARS-CoV-2 which has emerged as a global pandemic. In the absence of any confirmed drug moiety to treat the infection, the science fraternity adopted hit and trial methods to come up with a lead drug compound. This article is an overview of the virtual methodologies, which assist in finding novel hits and help in the progression of drug development in a short period with a specific medicinal solution.

## Plain language summary

An extensive survey of technological applications in drug discovery and development, encompassing offline and online approaches, is presented in this review. The span of research issues that can be tackled using these advances is vast, opening new horizons for future innovations. The article is designed to incite further research investments into drug development procedures and bridge existing research voids by outlining multiple pharmaceutical products that resulted from employing systematic computational methodologies.

## Tweetable abstract

The DDD process in pursuit of novel drug candidates is a challenging procedure requiring lots of time and resources. This article provides an overview of virtual approaches, which speedup the development of an effective drug molecule in a time effective manner.

## Graphical abstract

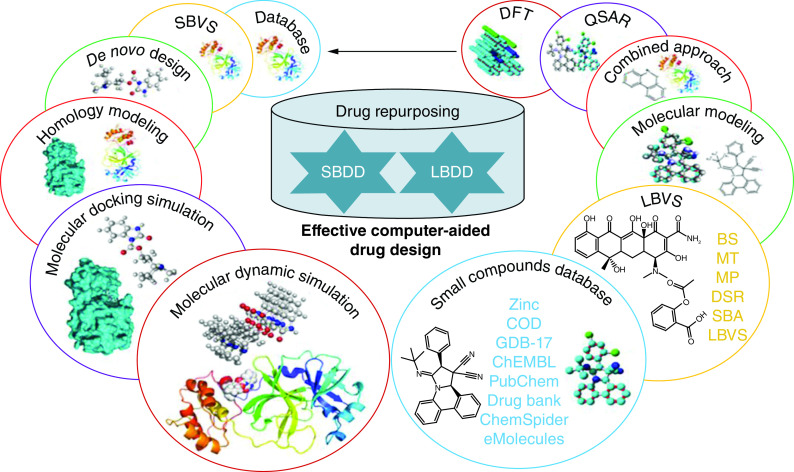

Many new diseases have surfaced in the last few years mainly due to drug resistance, changes in lifestyle, food contamination, and environmental pollution to name a few [1,2]. The current outbreak of severe acute respiratory syndrome (SARS) coronavirus (SARS-CoV-2) which has posed the biggest health concerns in the wake of 2020 is a recent example [3,4]. The viral outbreak was first reported from China where the increasing demand for animal proteins particularly from civets leading to their bearing in close confinements and in the absence of biosafety measures the virus spread like an epidemic from animals to humans [5]. What makes the virus so deadly is its rapid transmission from person to person which is very crucial for developing countries like India where the concept of joint families is very familiar with large number of people staying together under one roof and medical care facilities are still limited. In the absence of any concrete and specific medical solution, *in vitro* assessment and drug repurposing, US FDA-approved drugs on COVID-19 has been taken up and the drugs showed effectiveness [6,7] Figures 1 & 2.

**Figure 1.**
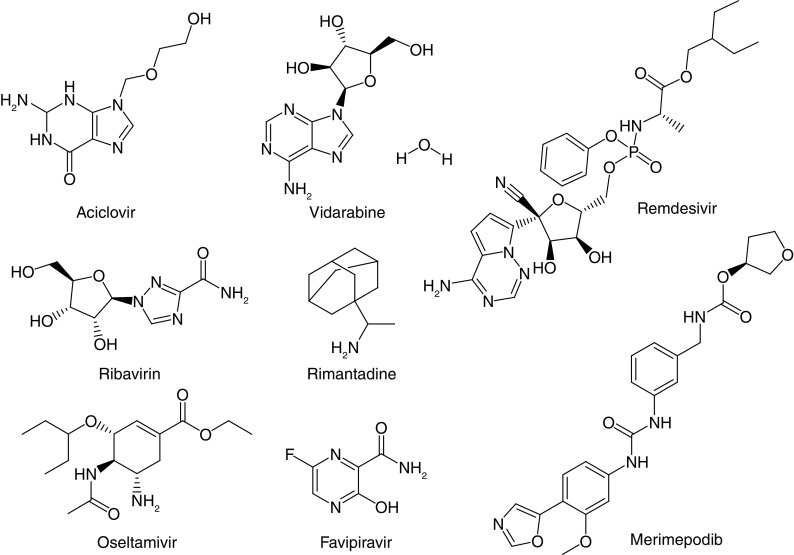
Known antiviral drugs.

**Figure 2.**
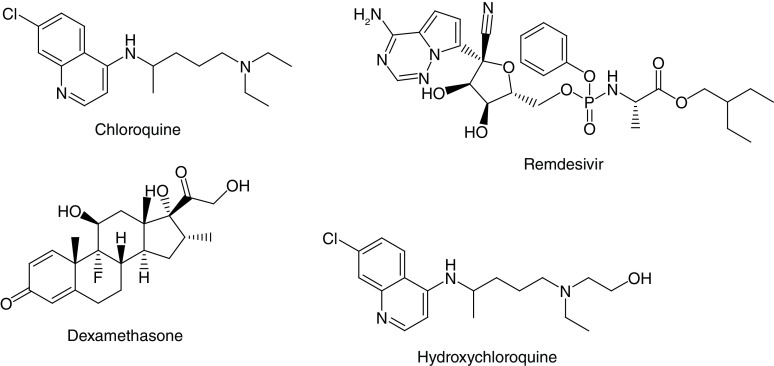
Well-known repurpose drugs against SARS-CoV-2 (COVID-19).

Combination therapy using different marketed drugs is also being explored, but no sure-shot confirmed drug has been achieved till now [8]. Since there is a growing necessity to combat the COVID-19 infection and future pandemics, a revolutionary change is needed in the pharmaceutical research to recognize the disease and develop effective medicinal remedies to combat new diseases [9]. The discovery of a drug, in general, requires a very high cost and time reaching up to 15 years. In many cases, drugs do not reach the market, because of the failure during clinical trials [10,11]. To overcome these problems, computational predictions have emerged as a boon for researchers [12]. Before the experimental study, the computational assessment provides key information needed for drug discovery [13]. Computational predictions can be made effortlessly with simple handling procedures [14]. ADMET (absorption, distribution, metabolism, excretion, and toxicity) parameters are the most significant initial factors that help in understanding various mechanisms like crossing physiological barriers and human intestinal absorbency, and with computational methods they can be predicted easily [15]. Molecular parameters, metabolic transformations, toxicity aspects, biological activity, and drug-receptor interactions can also be easily predicted by computational strategies [16]. Computational studies are based on extensive database information; currently, they are explored in the fields of molecular biology and chemistry to have an improved perceptive and integrated investigations such as in spectral analysis, molecular properties evaluation, protein sequence analysis, drug-receptor interactions, and interactions of genes [17,18]. Characterization of protein has also been realized using computational tools [19,20].

In this review, we have disseminated current advances and methodologies in pharmaceutical sciences and technology that are essential for drug discovery and development (DDD) including computer-aided screening (CAS), molecular docking, and molecular dynamic (MD) approaches as well as computer-aided drug design (CADD) that can deliver specific findings to expand the routes and methodologies in the development of novel drugs within a short period of time. We have deliberated upon the usefulness of CAS to overcome clinical therapeutic challenges. The crux of this review is to comprehend the complex virtual methodologies thoroughly. The main goal of this review article is to discuss all the available tools that are frequently used in drug discovery and development and can solve comprehensive research problems. This article is also very informative for researchers and readers in terms of problem-solving and future design. Additionally, it also discusses various drugs that were previously discovered by using different computation approaches separately in each section also motivates the researcher to contribute some more afford toward the drug development procedure and fill the research gap into it.

## Effective computer-aided screening

There are various advanced methods of developing molecular structures in drug design, discovery, and development. The well-known and conventional methodology is to study molecular structures with appropriate tools to draw discernments. Graphical exploration of evaluating protein–ligand interactions can provide information on the binding affinity of the ligands, such as, by adding a polar group that would be able to form a new hydrogen bond or a nonpolar group bringing it closer to the hydrophobic pocket [21,22]. Prediction of the interaction profile of the drug and receptor to illustrate the binding site additionally provides information about the diverse functional groups influencing the binding interaction [23,24]. For the identification of the appropriate ligand binding site, there are various tools available to identify the possible interaction sites in the receptor protein [25]. Similarly, protein-protein interactions can be computationally predicted for possible “hot spot” binding [26,27]. Furthermore, in the absence of a tentative structure of the ligand-receptor complex, molecular docking can be applied to identify ligand binding modes which contribute to the interaction analysis [28]. The flexible behavior of small molecules is typically explored by the docking algorithms, although the approximation of ligand binding affinity and the conformational flexibility of the macromolecule comprising alterations inside chains, key moieties, and changes upon ligand binding are more complex to find [29]. It is advisable to check various docking suites and investigate the reliability of the projected ligand binding modes [30]. If possible, the performance of a docking suite should be verified by control docking, in which the x-ray structure of the ligand from the ligand-receptor complex is re-docked to the crystal binding site and then the findings are related to each other to find the reliability of interactions [31].

The docking suite creates the most suitable molecular confirmations based on the empirical scoring function that assesses the released binding energy among the ligand-receptor interaction [32,33]. Meanwhile, the top-scoring outcome possibly does not reveal the definite binding pose due to the confines of scoring functions [34]. The area of ligand-receptor interaction is projected based on x-ray structures [35]. A broadly used technique in lead discovery is CAS. In this approach, compound libraries which can have millions of molecules and variable molecular systems are screened virtually to search for probable bioactive entities binding to a specific target. For instance, the analgesic effect of novel opioids with reduced side chains was identified from a library containing more than three million molecules through CAS approach [36,37]. Currently, CAS has emerged as one of the most efficient alternatives to high-throughput screening (HTS), which is also cost-effective [38].

The computer-aided screening starts by preparing the database or library of compounds, such as the conversion of 2D structures into 3D, preparation of tautomer, stereoisomers, and other possible conformers [39]. It is also worthwhile to pre-filter the compounds with the help of several drug character evaluation rules and filters like Lipinski rule of five (Ro5), Ghose and Veber, etc. to assess drug-like character including the oral bioavailability of the lead compound [40]. There are generally two types of computer-aided screening or virtual screening (VS) approaches, structure-based virtual screening (SBVS) and, ligand-based virtual screening (LBVS) [41]. SBVS method is reliant on the structure of the active site of macromolecules and the LBVS method is established for the evaluation of designed similarity among the identified active motifs [28,31]. In SBVS, it is essential to rationally select the macromolecule and the structure preparation is done by adding hydrogen atoms, cofactors, metal ions, or preserved water molecules in the ligand-binding site [42]. LBVS employs pharmacophore modeling whereas SBVS comprises docking and scoring of all the database molecules in the binding site of the macromolecule [43]. To avoid the ambiguity of scoring functions that rank the confirmation poses, one should execute the controlled docking and validate the results through diverse docking tools if possible [44]. When there are a number of active compounds, a reflective docking analysis can be executed to realize how well the CAS procedure can improve the identification of active compounds to the assembly of top-ranked compounds from a library of compounds [45]. To decrease the number of hits chosen, the best-ranked compounds can be additionally filtered on different parameters like chemical diversity, synthetic possibility, pharmacokinetics, pharmacodynamics, ADME, bioactivity score, toxicity, and metabolic transformation [46].

**Figure 3.**
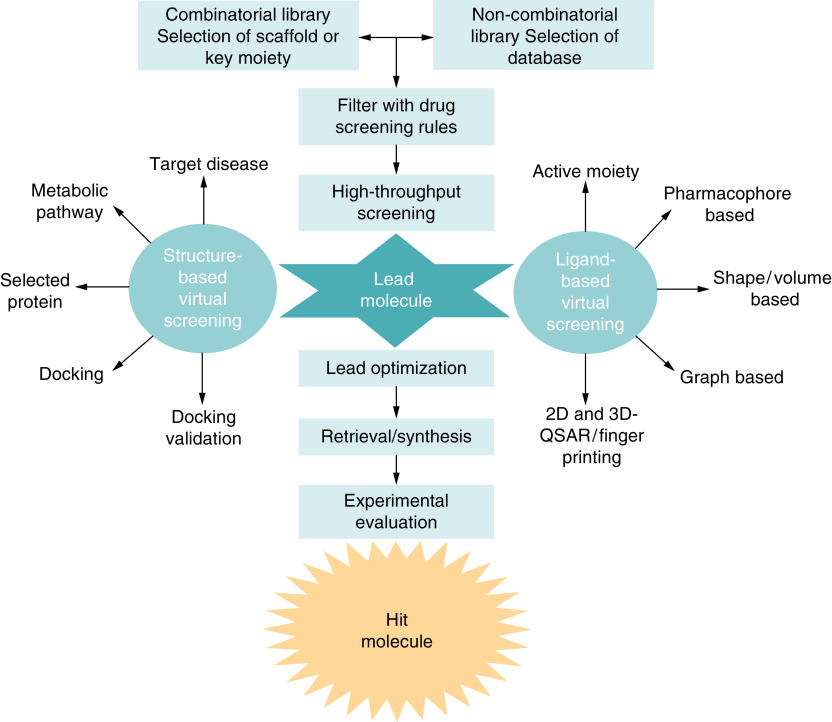
Schematic representation of computer-aided screening.

Quantitative structure-activity relationship (QSAR) is a ligand-based tactic frequently applied for lead optimization and assessment of compounds for synthesis/analysis [47]. The QSAR approach targets starting a quantitative correlation between the chemical structure of a molecular library and the experimentally observed biological activity (Interaction and inhibition of biological target) or other properties such as ADMET [48]. 3D-QSAR approaches also proceed through the interpretation of the 3D coordinates of the molecule and are extensively utilized in drug design (Figure 3) [49].

## Structure-based drug design tools

**Figure 4.**
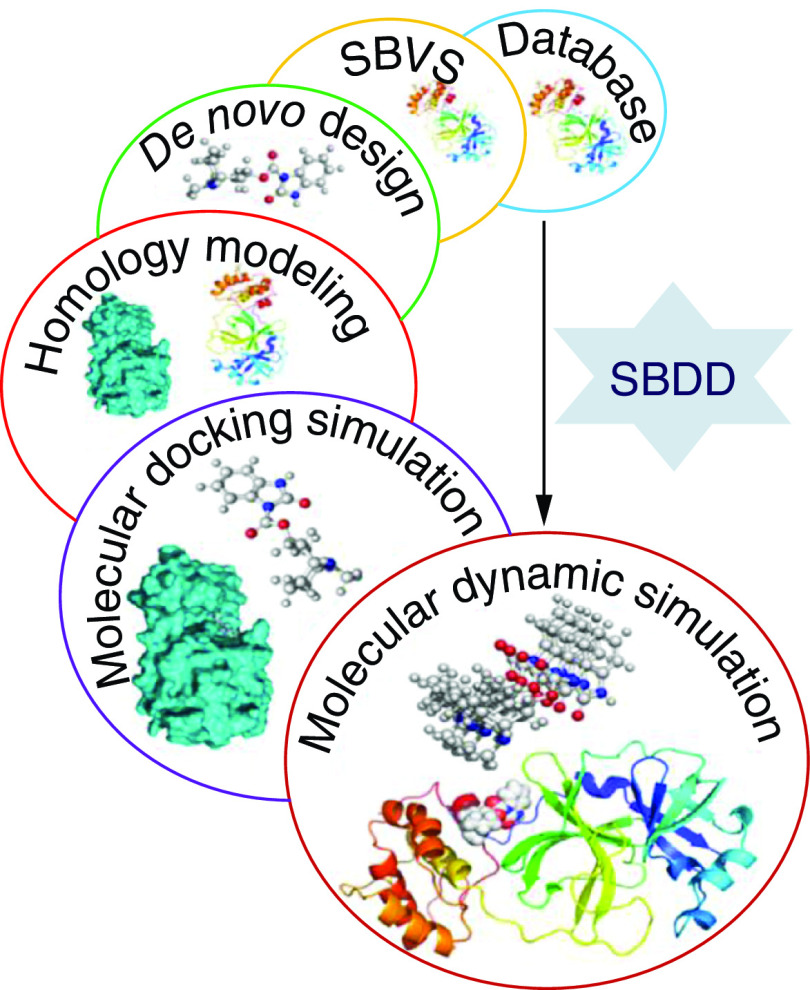
A simple pipeline of an effective structure-based drug design. SBDD: Structure-based drug design.

The structure-based drug design (SBDD) approach involves three-dimensional coordinates information, drug-likeness, and probable binding pocket [50]. Due to insufficient structural information obtained from experiments, a typical computer-aided approach including protein stringing methods and homology modelling (HM) can be used to obtain structural data [51,52]. HM utilizes a coordination model comparable to the protein to be modelled [53]. Process preliminary sequence alignment, is a typical task involved in HM. There are several Bioinformatic tools like COBALT [54], Kalign [55], Clustal Omega [56], and NCBI Blast [57], offering offline view and changes in the alignment which are critical, particularly when the investigator wants to know the exact position of the protein folds and the spheres that are required for additional modification. To estimate the target protein structure, the secondary structures are copied from the model constructed on the concluding sequence alignment [58]. The concluding model is optimized by minimization or MD analysis tools. Druglikeness enables modification of target receptors which should supposedly be done by a proposed drug candidate molecule [59]. A drug-likeness database is also openly accessible to permit investigators to submit binding side and druglikeness data. Binding site data should be attained from binding complexes with known ligands. A binding site is a hollow part of the protein that is classified by chemical topographies [60]. Nevertheless, if the binding site data is known, numerous computer-aided approaches are offered to recognize the possible protein binding pocket from a specified 3D structure [51], or a sum of evaluations can be utilized through blind docking [61], in which the complete receptor is taken as the interacting site, permitting ligand molecules to easily interact anywhere in the 3D coordinates of a receptor to find the most suitable interaction site. It is also notable that other possible binding sites (allosteric sites) may also be found on the protein surface. Conventional DDD frequently targets the main interacting site to block the substrate-binding site [62]. Nonetheless, it is found that ligands can also bind to the allosteric site and suppress the activity of the target protein in case of non-competitive inhibition. There are many case studies where the allosteric site is chosen as the main site to regulate the target protein (Figure 4).

**Figure 5.**
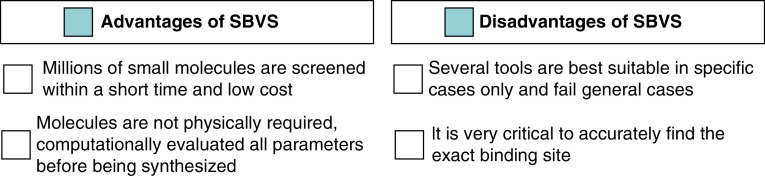
Advantages and disadvantages of structure-based virtual screening. SBVS: Structure-based virtual screening.

### Databases

In this epoch, big data have significantly affected the DDD. To take benefit of the CADD tools, these data are available for investigation by several open databases in cyberspace [63–66]. The furthermost, general, and broadly utilized protein data source is PDB and UniProt (https://www.rcsb.org/ and https://www.uniprot.org/). Genomic, and proteomic data for several targets like that of COVID-19 [67], can be obtained from UniProt. The clinical information about the patient can be sourced from https://covid19.who.int and https://datascience.nih.gov/covid-19-open-access-resources, which is a free program [68,69]. The eSLDB (eukaryotic Subcellular Localization database) is a protein subcellular localization database offered for the study of eukaryotes [70], PSORTdb for bacteria, and archaea database [71], and LOCATE for mammalians [72]. Drug repurposing and lead optimization are very helpful to explore the ligand-receptor interaction including Relibase, which involves ligand-receptor interactions [73]. BindingDB can be used to study the interaction between the drug candidate and the receptor [74], The protein-protein interaction profiles are evaluated with the Database of Interacting Proteins (DIP) [75], Biological General Repository for Interaction Datasets (BioGRID) [76], and Search Tool for the Retrieval of Interacting Genes/Proteins (STRING) [77]. Similarly, chemical databases are collections of organized, saved, and searchable chemical information and data. These databases provide details on chemical substances, their structures, reactions, spectra, and other pertinent information [78]. Chemical databases can be of use for professionals working in the field of chemistry, environmental science, material science, and drug discovery. To keep up with the most recent scientific advancements, these databases are often updated and expanded. Many databases also offer biological information (IC_50_ and Ki) of the scaffold library obtained from regular literature updates [78]. Their relevant details are summarized in Table 1.

**Table 1.** Free and commercially available databases of chemical libraries.

Database	Description	Size* (approximate)	City/country	Website link
PubChem	Small sized molecules along with bioactivity profile	103,000,000	New York/USA	https://pubchem.ncbi.nlm.nih.gov/
ChEMBL	Small sized molecules along with bioactivity profile	1,950,765	Cambridgeshire/UK	https://www.ebi.ac.uk/chembl/
ZINC	Small molecules	230,000,000	San Francisco/USA	https://zinc.docking.org/
SuperScent	Perfumes reported in literature.	23,000	Berlin/Germany	http://bioinf-applied.charite.de/superscent/
KEGG	Metabolites and small molecules	18,699	Kyoto/Japan	https://www.genome.jp/kegg/ligand.html
ChEBI	Small molecules	57,098	Cambridgeshire/UK	https://www.ebi.ac.uk/chebi/
COD (Crystallography Open Database)	Crystalloraphic structures of organic, inorganic, metallo-organics compounds and minerals	1,557,818	Cambridge/UK	http://www.crystallography.net/cod/
GDB-17	Molecules up to 17 atoms consisting of C, N, O, S, and halogens	166,443,860,262	Berne/Switzerland	http://gdb.unibe.ch/downloads/
DrugBank	Approved and investigational drugs	13,553	Alberta/ Canada	https://www.drugbank.ca/
PDBe	Ligands, monomers and small molecules	30,971	Cambridgeshire/UK	https://www.ebi.ac.uk/pdbe-srv/pdbechem/
BindingDB	Small molecules with bioactivity profile	820,433	San Diego/California	http://www.bindingdb.org/bind/index.jsp
ChemSpider	Small molecules	81,000,000	London/UK	http://www.chemspider.com/
eMolecules	Small molecules with bioactivity profile	5,900,000	La Jolla (CA)/USA	https://reaxys.emolecules.com/index.php

### Structure-based virtual screening

To identify the most suitable interaction profile between the receptor and ligand, SBVS is used as a promising approach. Based on the magnitude of interaction, they are ranked, and the topmost ranked ligand represents the closest interaction with the receptor. In SBVS three-dimensional structures of the receptor as well as the molecular library are required to evaluate their interaction profile [79]. Therefore, in this approach, a selected library of molecules is chosen from the databank and categorized based on their interaction with the receptor pocket. Molecular docking is a significantly used strategy to predict the extent of the interaction, due to its simple handling, quick response, and acceptable output [80]. The extent of interaction between the receptor and the ligand is validated through fitness score (FS) [81]. Some advantages and disadvantages of SBVS are represented in Figure 5. Recently, the SBVS approach has become a boon for the drug discovery process; due to the greater accuracy of the obtained results [82]. The following are the examples of a few drugs that were discovered using SBVS:

- Darolutamide (Nubeqa): used for the treatment of prostate cancer, was discovered in 2014 [83].
- Rucaparib (Rubraca): used for the treatment of ovarian and breast cancer, was discovered in 2003 [84].
- Vemurafenib (Zelboraf): used for the treatment of melanoma, was discovered in 2006 [85].
- Venetoclax (Venclexta): used for the treatment of chronic lymphocytic leukemia, was discovered in 2005 [86].

### *De novo* drug design

Artificial intelligence (AI) and deep learning techniques have brought revolutionary changes in CADD approach [87,88]. The use of these strategies in the design and development of novel drug candidates may lead to the development of a drug with selective properties like non-toxicity, and effectiveness against a specific receptor through automated generative processes [89,90]. A significant improvement is found in the novel *de novo* drug design process as a result of the combination of various protocols. Generally, two steps are involved in these processes [91]. The primary step uses huge, good-quality small compound databases to permit simulations to suggest discovering regulations related to the chemical scaffold. Generally, SDF, MOL, and SMILES files are used for the automated generation of a new scaffold [92]. It is helpful in the development of a new chemical scaffold based on the SMILES sequences and three-dimensional structure. The second step involves a reinforcement learning approach to accelerate the assessment of the scaffold-based space for finding of a new model [93].

#### *De novo* drug design methodology

**Figure 6.**
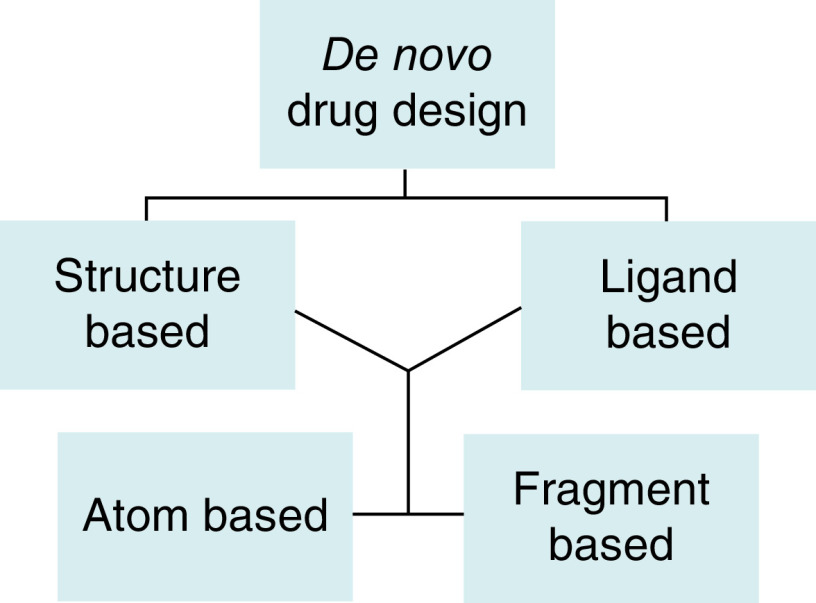
Schematic representation of the *de novo* drug-design methodology.

*De novo* drug design is a technique that develops new chemical compounds solely on the basis of a biological target (a receptor) or that target's known active binders (ligands found to possess good binding or inhibitory activity against the receptor). The construction of the molecules (sampling), evaluation of the generated molecules, and description of the receptor active site or ligand pharmacophore modeling are the main elements of *de novo* drug design [94]. There are two main methods for *de novo* drug design: structure-based and ligand-based. A receptor's three-dimensional structure can typically be determined via x-ray crystallography, NMR, or electron microscopy [95]. Homology modeling can be used to find a suitable structure for *de novo* drug creation when the receptor's structure is unknown. Yet, the qualities of the template structure and sequence similarity determine the quality of a homology model. The ligand-based method is typically employed when one or more active binders are known but no structural information for the biological target is available in Figure 6 [96].

*De novo* drug design is a method of drug development that entails creating a new chemical with a particular biological function. To create and improve new drug candidates, a variety of computational tools and approaches are applied during the process [97]. This approach's utilization of methodologies like linking and growth approaches is among its most crucial features. Using linking techniques, new molecules are produced by combining pre-existing scaffolds or fragments [95]. In this method, the scaffolds or fragments are chosen based on their known efficacy against the desired target. The fragments are linked in a way that maximizes the activity of the final molecule. On the other hand, growth methods entail the gradual assembly of a molecule from a small beginning unit. When there are no existing fragments that can serve as the basis for design, this method is often used. The target's known characteristics as well as the physical and chemical characteristics necessary for optimum activity serve as guidance for the growth process. *De novo* drug design's success depends on both linking and growth techniques [98]. They offer a methodical approach to chemical space exploration and the discovery of molecules with the best activity against a certain target. The possibility for developing novel medications using these techniques grows as computational technologies continue to progress. The software and webservers for *de novo* drug design using the classical approach are included in Table 2 for free and public use.

**Table 2.** Free and commercially available tools for *de novo* drug design.

Name	Mode	Description	URL
LigBuilder	Offline	A program for computer-aided drug design that includes docking and fragment growth algorithms.	http://www.pkumdl.cn:8080/ligbuilder3
OpenGrowth	Offline	Software for *de novo* drug design that is free, adaptable, and modular and employs the Monte Carlo method.	http://opengrowth.sourceforge.net
AutoGrow	Offline	Software for creating drugs that combines artificial intelligence with traditional programming.	https://durrantlab.pitt.edu/autogrow4
MolAICal	Offline	A program for structure-based *de novo* drug design with multiple applications.	https://molaical.github.io
e-LEA3D	Online	A web server tool that uses a traditional algorithm and artificial intelligence to assist in drug creation	https://chemoinfo.ipmc.cnrs.fr/LEA3D/index.html
WADDAICA	Online	A web server tool for combinatorial library design, virtual drug screening, and *de novo* drug design	https://bqflab.github.io

### Homology modeling (HM) in drug discovery

HM is the most acceptable computer-aided scaffold-based prediction approach. It is broadly utilized to explore the three-dimensional structure of receptors from the sequence of amino acids. HM is a combination of various phases and there are numerous tools that are freely available and broadly used for HM [99]. Every available tool has its own characteristics. Thus, the quality of the HM is based on the condition of the produced three-dimensional structure of the receptor [100]. HM offers a number of applications such as in search of drug-receptor interactions, the knowledge of the exact three-dimensional structure of the receptor is essential that can be modeled by HM. Therefore, HM has become an essential approach in search of a new drug candidate. This approach has been used successfully in the development of several commercial drugs. Here are a few examples:

■ Darunavir is a protease inhibitor used in the treatment of HIV infection. It was developed using homology modeling to predict the structure of the drug target, the HIV-1 protease, based on the known structure of a related protease from another virus [101].
■ Gefitinib is a tyrosine kinase inhibitor used in the treatment of non-small cell lung cancer. It was developed using homology modeling to predict the binding of the drug to the ATP-binding site of the target protein, the epidermal growth factor receptor [102].
■ Axitinib is a tyrosine kinase inhibitor used in the treatment of renal cell carcinoma. It was developed using homology modeling to predict the binding of the drug to the ATP-binding site of the target protein, the vascular endothelial growth factor receptor [103].
■ Sitagliptin is a dipeptidyl peptidase-4 inhibitor used in the treatment of Type 2 diabetes. It was developed using homology modeling to predict the interaction of the drug with the catalytic site of the target protein, dipeptidyl peptidase-4 [104].
■ Overall, homology modeling has been a valuable tool in drug discovery, allowing researchers to design drugs that specifically target proteins involved in disease pathways.

### Molecular docking simulation

Critical molecular mechanisms, ligand binding approaches, and factors influencing the drug-receptor interactions can be estimated with the help of the docking results [105]. Docking suites can be used to calculate the binding energies associated with the most stable conformation of drug-receptor interactions [106,107]. Docking analyses are carried out to optimize the interaction pattern among the ligand and target and to find the best confirmation of the ligand in the docking complex with the help of free binding energy [108]. Empirical scoring functions are investigated that convert free binding energy into the docking score [109]. To create a 3D pose, and ligand, and target interaction patterns, a variety of free online programs are offered, including Biovia DSV, Pymol, Chimera, Rasmol, and Swiss PDB viewer, etc. Two different docking types are generally used, which are discussed below:

#### Rigid docking

The primary geometry of the target and ligand is preserved during the docking analysis in rigid docking. This kind of docking analysis is based on Emil Fischer's 1894 “lock and key” concept [110]. To study how drugs interact with receptors, ligand-target docking analysis is required. However, there is an issue when a ligand is docked at a protein's pocket. The rigid structure of both makes it difficult to observe the interaction and to find the best confirmation of the ligand [111]. Sometimes ligands fail to bind to a protein's pocket, resulting in a weak interaction that does not provide the desired results. Good docking contact requires internal flexibility. The structural alterations upon binding can occasionally be so slight that rigid docking is required to see the interaction [112]. The ease and speed are further advantages of rigid docking protocol.

#### Soft docking

Target and ligand side chains are both kept flexible during soft docking. The induced-fit concept put forward by Daniel Koshland in 1958 serves as the basis of flexible docking. The free binding energies of various conformations of the suggested ligand are computed at the pockets of a target [112,113]. Moreover, the target chain is adaptable enough to integrate ligand interaction with target conformational alterations. The most widely used and accurate method can forecast a wide range of potential changes in the ligand's structure, but it is also time-consuming and expensive [114].

#### Docking algorithms

The most appropriate structural position with the lowest free binding energy is provided after accounting for all conceivable conformations of the ligand under study during its interaction with the receptor target [115,116]. The most often exploited docking analysis methods are:

- Molecular dynamics
- Monte Carlo methods
- Genetic algorithm
- Fragment-based methods
- Point complementary methods
- Distance geometry methods
- Systematic searches

#### Types of energies evaluated

To get the lowest free binding energy during the ligand-target interactions, docking analysis' primary goal is to find the optimal conformation of the ligand. Molecular docking software typically calculates the scoring functions of a ligand-target interaction [112]. The final binding energy (ΔG bind) is determined as a sum of various energies, including the H-bond, torsional free, electrostatic, desolate energy of the unbound system, total internal, dispersion, and repulsion, among others. The binding energy is denoted by the dissociation constant (K_d_) in terms of Gibbs' free energy (ΔG). The same variables, including intermolecular interactions, desolation, and entropic effects, influence the prediction of drug-receptor binding. The scoring function's efficiency raises together with the assessment of the physiochemical parameters [117]. The empirical scoring function of any docking program is given as Equation 1 and the binding energy is given as Equation 2.

(Equation 1) Fitness = vdW + H bond + Elec

(Equation 2) $$\Delta {\text{G}}_{\text{bind}}\;=\;\Delta {\text{G}}_{\text{vdw}}\;+\;\Delta {\text{G}}_{\text{hbond}}\;+\;\Delta {\text{G}}_{\text{elect}}\;+\;\Delta {\text{G}}_{\text{conform}}+\;\Delta {\text{G}}_{\text{tor}}\;+\;\Delta {\text{G}}_{\text{sol}}$$

#### Common docking programs

##### AutoDock

The Molecular Graphics Lab at the Scripps Research Institute in La Jolla, California, USA, established the open-source AutoDock docking tool. The estimation of the binding site of biological macromolecules and ligands can be done successfully using it [115]. The formula provided in the form of the scoring function is the foundation for the binding energy computation. The AMBER force field and linear regression analysis utilizing the Lamarckian genetic algorithm (LGA) are used to establish the AutoDock score function [118]. It focuses on evaluating ligands for docking reinforcement using nearly 0–10 flexible bonds. The interaction pattern of a drug-like candidate is commonly explored using the exceptionally effective default parameters of AutoDock. Additionally, it is widely employed for virtual screening and to obtain frequently docked conformations of the ligand concerning a target. For instance, drug-receptor docking, protein-protein docking, molecule optimization, analysis arising from structure-based drug design, validation of the action mechanism of drug molecules, etc. are only a few applications of AutoDock.

##### AutoDock Vina

In the Molecular Graphics Lab at the Scripps Research Institute, AutoDock Vina was developed by Oleg Trott in 2010 [119]. It exhibits good efficiency, multi-core competency, improved accuracy, and a straightforward handling technique. It is a relatively new, easily accessible tool for molecular docking, drug development, and virtual screening. Vina independently forecasts the clusters and grid maps and significantly improves the precision of the computations related to AutoDock's interaction mode. When compared with other techniques, Vina has been able to predict outcomes more accurately [120].

##### AutoDock FR

Dr. Pradeep Anand Ravindranath developed AutoDock FR (ADFR: AutoDock for Flexible Receptors) in the Integrative Structural and Computational Biology Lab at the Scripps Research Institute in 2015. ADFR was developed to investigate the interaction between target proteins and small, flexible ligands. It enables the flexible creation of target protein side chains to mimic the induced fit [121]. The side-chain flexibility of up to 14 targets is controlled by ADFR, and the proficient growth of the docking realization rate is over 50%. Up to 12 flexible receptor sidechains on the cross are investigated through docking. Results from ADFR are often more accurate than of AutoDock Vina. By progressively increasing the number of flexible receptor sidechains, Vina demands an arbitrary run time for docking; in contrast, ADFR needs linear run-time adjustments [121].

##### iGEMDOCK

The Institute of Bioinformatics at National Chiao Tung University in Taiwan developed the iGEMDOCK tool for docking, drug design, screening, and post-screening analysis. It is a multifunctional automatic graphics application [122]. As in AutoDock, iGEMDOCK coordinate files are prepared by including torsions, bond order, hydrogen atoms, and charges. Both the ligand and the target are assigned these parameters. The input and output files for iGEMDOCK are PDB and Mol respectively. The most appropriate conformation of the ligand is automatically chosen by iGEMDOCK, which also provides the total binding energy [122]. The fitness score used to calculate iGEMDOCK scores is indicated as Equation 3.

(Equation 3) The fitness score = Van der Waal energy + Hydrogen bond energy + Electro-statistic energy

The estimate of target binding sites and structural optimization are crucial during the docking assessment. The scoring function is significantly impacted by hydrogen bonds present in the docked complex. In addition, internal H bonds and electrostatic interaction are predicted as sp^2^-sp^2^-torsions from the interaction complex, which can significantly reduce the total number of suspected H bonds. The generic evolutionary approach (GA) offers three efficient docking techniques *viz*. standard docking, stable docking, and accurate docking-iGEMDOCK functions. With a maximum of 80 cycles or generations, an 800-population size, 8000 interactions, 10 numbers for the solution, and 100 energy thresholds available, accurate docking is a highly slow docking methodology. The hydrophobic and electrostatic affinities are adjusted to 1.00 for excellent efficiency. Minimum rotations, translations, and torsion checks are performed for each step. Usually, iGEMDOCK chooses the conformation with the lowest energy.

A positive energy value is found when iGEMDOCK estimates an electrostatic interaction that is not favorable. If the docked pose is more related to the specified ligands than the RMSD threshold, set the threshold and add energy penalty (i.e., the 100-energy penalty, the 2.00 RMSD threshold, and atom ID (quick) RMSD calculations were all established.) into the scoring function, the docking program resolves and emphasizes its prior investigation and identifies their variants. When ligand-target docking is finished, binding affinities (kcal/mol) and docking run time are provided as the lowest binding energy conformation. The lowest binding energy conformation is automatically chosen as the best result. Comparing iGEMDOCK to certain other docking programs, its overall docking efficiency is easier and superior (Table 3).

**Table 3.** Server/software of the molecular docking analysis.

Server/ software	Availability	Developer	City/country	Web link
AutoDock 4	Free standalone program	The Scripps Research Institute	La Jolla, (CA)/USA	http://autodock.scripps.edu/
AutoDock Vina	Free standalone program	The Scripps Research Institute	La Jolla, (CA)/USA	http://vina.scripps.edu/
iGEMDOCK	Free/open-source platform	BioXGEM Lab. Institute of Bioinformatics National Chiao-Tung University	Hsinchu/Taiwan	http://gemdock.life.nctu.edu.tw/
DOCK	Free/open-source platform	University of California, San Francisco	San Francisco /USA	http://dock.compbio.ucsf.edu/
GOLD	Commercially available	The Cambridge Crystallographic Data Centre	Cambridge/USA	https://www.ccdc.cam.ac.uk/solutions/csd-discovery/components/gold/
Glide	Commercially available	Schrödinger, LLC	New York/USA	https://www.schrodinger.com/glide
FlexX4	Commercially available	BioSolveIT GmbH	Sankt Augustin/Germany	https://www.biosolveit.de/FlexX/
SwissDock	Free webserver	Swiss Institute of Bioinformatics	Lausanne/Switzerland	http://www.swissadme.ch/
CDOCKER	Commercially available	BIOVIA	San Diego/California/USA	https://www.3dsbiovia.com/
Pharmer	Free/open-source platform	Department of Computational Biology, University of Pittsburgh	Pittsburgh/Pennsylvania/USA	http://smoothdock.ccbb.pitt.edu/pharmer/

#### Use of molecular docking

In the last decade, technologies have been regularly updated like high-throughput sequencing, and x-ray crystallography and the crystal structures of large numbers of proteins have been defined. Consequently, the structural and functional significance of biological macromolecules (like protein/enzyme) has been expanded and many novel drug targets have been identified [123]. Due to the revolution of computational science in various fields of research the utilization of virtual screening and molecular docking in DDD has been significantly stimulated [124]. Currently, computer-aided technology has become a key tool in DDD, via molecular docking simulation the analysis of the mutual interaction of drug-receptor becomes very easy along with large accuracy and boost the drug development procedure by reducing the period [125].

Reverse molecular docking takes into account the library of small molecules as a key structure to execute molecular docking in the spatial or 3D target database and evaluate the conceivable larger entities to conclude the three-dimensional structure and energy of identical assessment meaning that it identifies the most suitable target with minimum binding energy. For that reason, the development of reverse molecular docking delivers a fresh route to discover the suitable target of the drug compound and reveals the drug action mechanism [126].

### Molecular dynamics simulation

Molecular dynamics (MD) simulations are the most extensively utilized technique for the analysis of the conformational flexibility of small molecules, proteins, and other biochemicals [127]. In the search of a novel therapeutic agent, MD is used to explore the conformation evaluation of small molecules that can assist the DDD process. Moreover, MD can significantly contribute to the search for alternate conformations of a drug candidate and help in the identification of the temporary pocket point of the protein-protein crossing site. It also directly analyses the consequence of a single nucleotide polymorphism (SNP) on target protein–ligand interactions [128]. Currently, MD has utilized filtering models of biomacromolecules and observed uncertain stability laterally with docked protein–ligand complexes. Conversely, MD simulation is capable of vigorous assessment as well as boosting extra transparency in the observation [129]. Estimation of the free binding energy of the bound ligands is also assessed with the help of MD but still, it needs modification and observation, such as massive conformational variants associated with the collected proteins. Overall, molecular dynamics has played a significant role in the discovery and development of commercial drugs by providing insights into the dynamic behavior of biological molecules and the interactions between drugs and their targets. Here are some examples of drugs that were discovered or improved using molecular dynamics:

■ Zanamivir is an anti-viral drug used to treat influenza. It was discovered using molecular modeling techniques that predicted the optimal structure of the drug for binding to its target enzyme, the neuraminidase protein. This led to the development of a potent and specific inhibitor of this enzyme [130].
■ Darunavir is an anti-HIV drug that was developed using molecular dynamics simulations to optimize its binding to the HIV protease enzyme. By understanding the dynamic behavior of the enzyme and its interactions with the drug molecules, scientists were able to design a drug that is more effective and less prone to resistance [101].
■ Imatinib is a targeted cancer drug that was developed by modeling the interaction between the drug and its target kinase enzyme, BCR-ABL. This process involved molecular dynamics simulations to examine the stability of the drug-enzyme complex and identify key residues for drug binding [131].
■ Albuterol is a bronchodilator used to treat asthma and COPD. The design of this drug was informed by molecular dynamics simulations that helped to elucidate the structural and dynamic properties of the drug and its target receptor, beta-2 adrenergic receptor. This led to the development of a more specific and potent drug with fewer side effects [132]

#### Essential models in MD simulation

##### Pivotal cooperative role

In MD simulation, the dynamic interaction between individual atoms and their surroundings is predicated on the notion that every atom in the system interacts with its nearby atoms in a complex web, which together determine the overall behavior and stability of the system. The fundamental cooperative role model can be visualized as a sort of “dance” between the simulated atoms as they continuously modify and adapt to their environment. Electrostatic interactions, van der Waals forces, and chemical bonding control the overall shape, structure, and characteristics of the system. In the end, the important cooperative role plays a crucial part in many cutting-edge applications, spanning from drug discovery and materials research to biophysics and biochemistry, and is crucial for understanding the behavior of complex molecular systems [127].

##### Periodic boundary conditions

Individuals simulate a minor measure of the real material and manufacture the model coordination of a vast molecular system by the equivalent assets. This fragment occurs periodically in 3D space and signifies the entire system.

##### Assimilation period extent

The straightforward impression of MD prediction is to precede the usual motion of the molecule to excerpt the model in the level space for arithmetical prediction later benevolent the preliminary motion phase of the molecular system. The assimilation period is the test group rest. The belief in picking the suitable assimilation period extent is that the assimilation period extent is less than 1/10 of the fastest motion epoch in the system. It reduces time, confirming the accuracy of the prediction.

##### Probable task

The probable task or potential function is a function relating the relations among molecules. The interaction concerning molecules switches the interface activities, flanked by the molecules, which essentially regulates each property of ingredients. This consequence is exactly defined by the probable task. In MD simulation the choice of the probable task shows a significant character in the simulation.

#### General ethics of MD simulation

##### Use of MD simulation

The use of MD simulation has rapidly increased. For instance, some researchers applied MD to simulate the melting process of small-sized metal clusters and used it to connect with inserted multi-core perspective to simulate the heating and melting process of Ni nanoclusters [133,134]. Similarly, MD simulation along with quantum chemical calculation procedures were used to evaluate diverse ionic liquids attained the fundamental characteristic, spectral characteristic (UV-Vis, IR, Raman, and NMR spectroscopy, etc.), and catalytic reaction mechanisms of ionic liquids (Table 4).

**Table 4.** Popular server/software of the MD simulation.

Database/software	Availability	Developer	City/country	Web link
GROMACS	Free/open-source platform	University of Groningen and Uppsala University	Groningen/TheNetherlands, and Stockholm/Sweden.	http://www.gromacs.org/
AMBER	Commercially available	University of California, San Francisco	CA/USA	https://ambermd.org/
BOSS	Commercially available	Jorgensen Research Group, Yale University	New Haven (CT)/ USA	http://zarbi.chem.yale.edu/software.html
NAMD	Free/open-source platform	University of Illinois at Urbana–Champaign	Champaign/ USA	https://www.ks.uiuc.edu/Research/namd/
LAMMPS	Commercially available	Sandia National Laboratories Temple University	Albuquerque, New Mexico and Livermore (CA)/USA	https://lammps.sandia.gov/
CHARMM	Free or commercially available	Martin Karplus, Accelrys	San Diego (CA)/USA	https://www.charmm.org/
SwissParam	Free webserver	Swiss Institute of Bioinformatics	Lausanne/Switzerland	http://www.swissadme.ch/

#### General procedure of MD simulation by GROMACS

MD simulation is a computational method used to examine the atomic level dynamics and behavior of molecular systems. The popular software program GROMACS can be used to carry out these simulations. The basic steps for setting up and executing an MD simulation with GROMACS are as follows:

■ Preparation of the system: prepare the protein–ligand complex using molecular modelling software such as PyMOL or VMD. Next, solvate the system in a box of water molecules and add counterions to neutralize it. Save the system in .pdb or .gro format in the working directory.
■ Parameterization: create a topology file that describes the force field parameters of the system. GROMACS provides several force fields like GROMOS, CHARMM, and AMBER that can be used for the simulation. You need to choose a force field for the simulation based on the desired system.
■ Energy Minimization: run an energy minimization step to relax the system and remove any unfavorable contacts or bad geometry. This is done using the steepest descent and conjugate gradient methods.
■ Equilibration: perform a series of equilibration steps to allow the system to adapt to the simulation conditions. These steps involve gradually increasing the temperature, pressure, and density of the system over time.
■ Production simulation: run the production simulation to generate the required conformational changes and ascertain important properties that need to be studied. Save the trajectory in a .xtc or .trr file format.
■ Analysis: analyze the trajectory and calculate properties like root-mean-squared deviation (RMSD), root-mean-squared fluctuation (RMSF), radius of gyration (Rg), and hydrogen bonding using GROMACS tools like g_rms, g_rmsf, g_hbond, etc.
■ Visualization: finally, view the simulation results using software such as VMD, PyMOL, or Discovery Studio to visualize and interpret the results [135,136].

Note: A basic set of instructions for utilizing GROMACS to execute an MD simulation is provided above. Depending on the system being investigated and the research topic being addressed, different steps and parameters will be used.

## Ligand-based drug discovery tools

**Figure 7.**
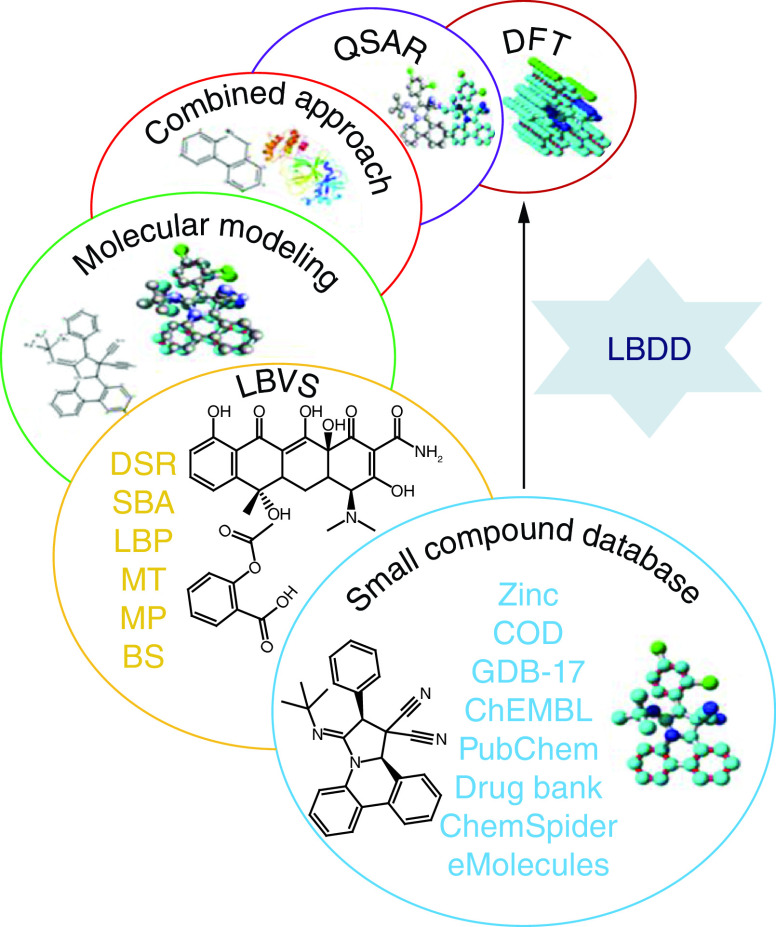
A simple pipeline of effective ligand-based drug discovery. COD: Crystallography Open Database; DFT: Density functional theory; LBDD: Ligand-based drug discovery; LBVS: Ligand-based virtual screening; QSAR: Quantitative structure-activity relationship

Ligand-based drug discovery (LBDD) is initiated along with a chemical scaffold or their library potentially active against a particular receptor because of SAR information, the effectiveness, and other essential assets of the chemical scaffold are enhanced by developing suitable analogs [137]. Topliss method and modest analogs designing can be a very proficient model based on scaffold resemblance [138]. Therefore, for design purposes, computer-aided approaches, including pharmacophore or three-dimensional models can be valuable. When a huge set of data with significant diversity is prepared, QSAR approaches can be applied [139]. Likewise, when a group of chemical scaffolds that are previously identified as effective against a particular receptor through databases and literature, then a computer-aided approach can also be utilized [140]. The computer-aided approaches are strongly adequate for sorting scaffold leads, virtual screening, and design proposals (Figure 7).

### Small compound databases

Recently in cyberspace, a huge expansion of small compound databases is found and significantly contributes to the CADD. Now, millions of small compounds are accessible in openly available databases [141,142]. Several databases additionally provide biological data (IC_50_ and Ki) on scaffold libraries derived from routine literature updates; their pertinent details are compiled in Table 1.

### Ligand-based virtual screening

#### Drug screening rules

**Figure 8.**
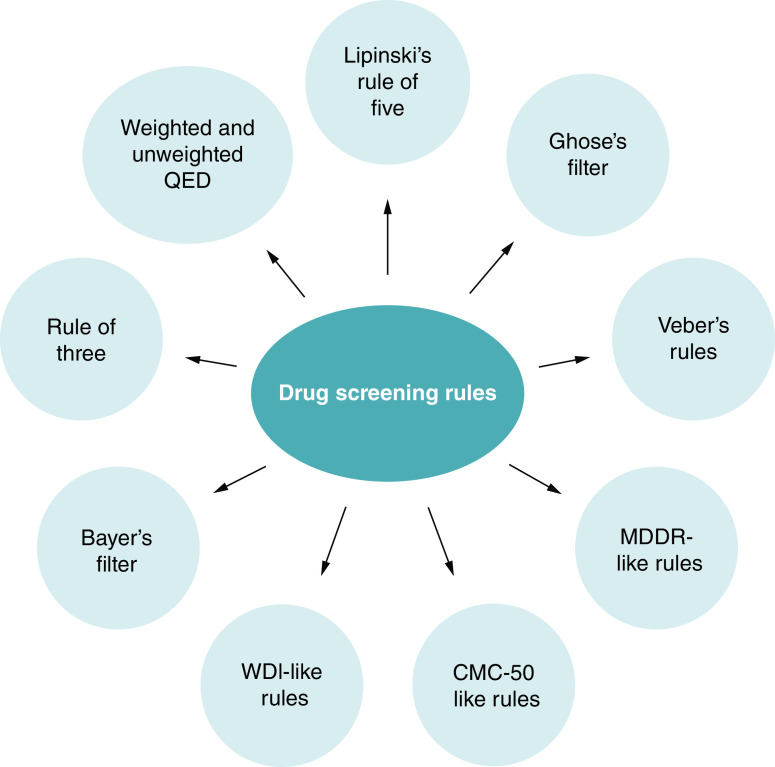
Drug screening or filtering rules.

Based on available drug molecules in the database, researchers have proposed some rules which predict the drug character in novel molecule-based parameters including the number of rotatable bonds (RB), cLogP, molecular weight (MW), number of hydrogen bond donor (HBD or NHOH), and acceptor (HBA or ON), etc [143–145]. (Figure 8 and Table 5).

**Table 5.** Drug filtering rules.

Molecular properties												
Rules	MW (Da)	TPSA (Å^2^)	HBD	HBA	Rot.B	logP	MR	Rigi.B	Atoms (n)	Rings (n)	Carbon atoms (n)	Hete. atoms (n)
Lipinski's rule of five	≤500	–	≤5	≤10	≤10	≤5	–	–	–	–	–	–
Ghose's filter	160–480	–	–	–	–	-0.4–56	40–130	–	20–70	–	–	–
Veber's rules	–	≤140	≤12	0 ≤12	≤10	–	–	–	–	–	–	–
MDDR-like rules	–	–		–	≥6	–	–	≥18	–	≥3	–	–
CMC-50 like rules	230–390	–	–	–	–	1.3–4.1	70–110	–	30–55	–	–	–
WDI-like rules	12–550	–	5–12	9–20	8–13	5–10	8–120	–	–	–	–	–
Bayer's filter	200–600	≤150	≤10	≤5	≤15	2–5	–	–	–	≤7	>4	>1
Rule of three	<300	≤60	≤3	≤3	≤3	–	–	–	–	–	–	–
Lead-likeness	<350	–	–	–	–	<3	–	–	–	–	–	–
Brenk	–	–	<4	<7	<8	0–4	–	–	–	<5	–	–

MW: Molecular weight; TPSA: Topological polar surface area; HBD/nOHNH: Hydrogen bond donor; HBA/nON: Hydrogen bond acceptor; Rot.B: Rotatable bonds; LogP: Logarithm of compound partition coefficient between n-octanol and water; MR: Molar refractivity; Rigi.B: Rigid bonds; Hete.: Hetero atoms.

#### Molecular & pharmacokinetic parameters

##### Molecular weight

Molecular weight (MW) is one of the most effective parameters for the evaluation of druglikeness and represents the activity of a molecule in the actual biological system. Large molecules have difficulty crossing over the biological membranes [146]. Furthermore, in the absorption of a drug molecule, it also plays a key role. Researchers have reported that 80% of the known drugs have MW within 450 Da [147].

##### Topological polar surface area

The calculated polar surface has been associated with the total number of hydrogen bonds and a total number of hydrogen bond donors and acceptors. The Polar surface area and hydrogen bonds increase with the increase in molecular weight indicating a correlation [146]. Veber *et al.* described that reduced PSA is related to a higher penetration rate [148]. The impact of the high polar surface area has been associated with intestinal absorption. According to Veber's rule, molecules having PSA values equal to or less than 140 Å^2^ exhibited good oral bioavailability in rats [149].

##### Hydrogen bonds (HB)

**Figure 9.**
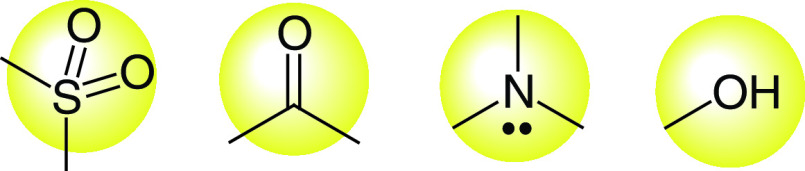
Most biologically active function groups.

Hydrogen bonding has a vital significance in biological systems. During the process of hydrogen bond formation among the donor and acceptor atoms in a natural system [150], molecules also form hydrogen bonds with the surrounding water molecules. Many drugs are absorbed orally through the gut wall by *trans*-cellular absorption, but when molecules form multiple hydrogen bonds along with the water molecules, it causes a delay in the movement of drug molecules from the gut into the blood (Figure 9) [151,152].

According to Muegge *et al.*, four groups represented in Figure 3 are distinct for exhibiting drug-likeness including hydrogen-bonding capacity which is necessary for definite drug interactions with specific targets [153]. The presence of some other functional groups including amidine, carboxylic acid, urea, amide, carbamate, amine, guanidine, sulfone, alcohol, sulfonamide, ketone as well as ester also influence the ability to form hydrogen bonds [154].

##### Rotatable bonds

Veber *et al.* described a rotatable bond as a single bond without any terminal heavy atom which does not belong to the ring [148]. Flexible drug molecules have many conformational limitations as well as the loss of entropy upon binding (∂G =∂H-T∂S). On the other hand, in a rigid molecule, a minimum loss of entropy is observed. Approximately 65% of the discovered drugs have partial rigid character and have good to excellent oral bioavailability [155–157].

##### Molar refractivity

The main significance of molar refractivity (MR) is in the depiction of the polarizing power of a molecule in a particular material [158]. MR is a chemical property that is associated with the structure of the molecular system and generally, the Lorenz-Lorentz Equation 4 is used to calculate it [159].

(Equation 4) $$\text{MR}=\left[\frac{{\text{n}}^{2}-1}{{\text{n}}^{2}+2}\right]\left(\frac{\text{MW}}{\text{r}}\right)=1.333\;\text{pNa}$$

Where; MW = molecular weight

(MW/r) = molar volume

N = molar volume

a = polarizability

n = refractive index

r = density

The equation 4 applies to both the solid and liquid states of the system. MR is linked with the volume of the molecules as well as to the immediate dipole-induced dipole forces or vdW forces that arise during drug-receptor interactions [158]. MR is dependent on the MW, refractive index, and density of the bulky substances and predicts the binding ability and lipophilicity of the concerned molecule. It is very helpful in the analysis of the quantitative structure-activity relationship (QSAR) of the molecule [160,161].

##### cLogP

cLogP is applied for the measurement of the partition coefficient of the proposed molecule between water and n-octanol. It is calculated by the following formula-

cLogP = [X]Octanol/[X]aqueous

Molecules with negative cLogP are unable to cross cell membranes and hardly enter the hydrophobic interior of the lipophilic bilayer [162]. cLogP represents the solubility of the proposed compounds affecting the capability of a molecule to get via the cell membrane [163]. The most accepted value of cLogP is within the limit of 5.0. Certain researchers have described the association of cLogP with blood–brain barrier (BBB) penetration. cLogP is considered the most significant factor for a drug molecule to exhibit CNS activity. Researchers have reported that the value of cLogP should be around 4–5 for a CNS active compound [164].

##### Rigid bonds

The drug molecules should be more rigid and have the essential chiral complexity to interrelate with the residues in binding sites to fill more entirely the existing space on the site and to avoid target protein flexibility. Rigidity also ensures that the drug compound would not change during the chemical interaction to avoid any undesirable interaction [165].

##### Number of atoms

The number of atoms has a significant role as they are directly linked with the MW, PSA, cLogP, and other pharmacokinetic parameters. In a potential drug candidate, the number of atoms must be between 20–70, according to Ghose's filter, and 30–55 based on CMC-50-like rules [166–168].

##### Number of rings

No more than two fused rings are suitable for a target drug candidate, according to the literature. Some rings are also associated with the surface and binding properties of the molecular system [169–171].

##### Number of carbon atoms

Muegge *et al.*, found that an ideal drug must have more than four carbon atoms. A carbon atom tends to bond with four different functional groups leading to chirality [153]. Nitrogen, sulfur, and phosphorus can also show chirality like in methaqualone, omeprazole, and cyclophosphamide [172]. Chiral compounds show optical activity [173]. Presently, more than half of the marketed drugs are chiral. Chirality in the molecular system promotes biological as well as pharmacological activities (ADMET) [174,175].

##### Number of hetero atoms

**Figure 10.**
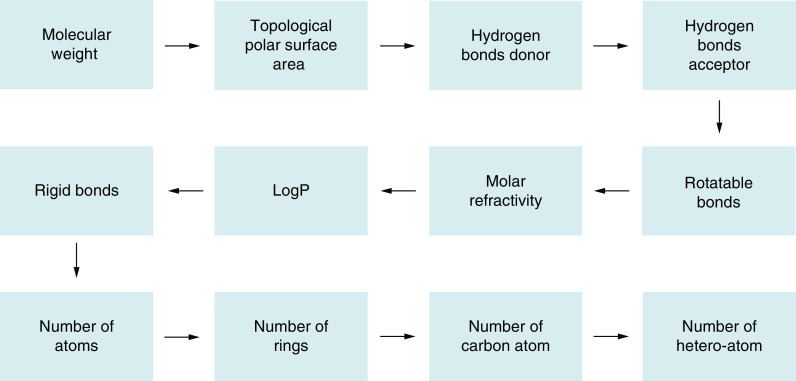
Drug screening molecular parameter based on drug filtering rules.

Heterocyclic compounds have a minimum of one heteroatom in their structure [176] and are widely applicable in the files involving pharmaceuticals, agrochemicals, and used as veterinary agents. Certain natural products including antibiotics (penicillin and cephalosporin) and alkaloids (vinblastine, morphine, and reserpine) have heterocyclic entities [177,178]. Recently, they have found utility as sanitizers, corrosion inhibitors, developers, copolymers, antioxidants, and dye, and additionally as a carrier in the synthesis of organic compounds. According to Bayer's filter, a good drug candidate must have more than one hetero atom (Figure 10).

##### Structure accessibility

Structure accessibility (SA) plays a dynamic role in drug design and discovery [179]. Evaluation of synthetic accessibility of the molecular system before the wet lab stage is of utmost significance [180]. Assessment of SA of a bulky chemical moiety is an enormous task. Therefore, to predict SA within a short period, several computer-aided techniques have been developed. Currently, a simple method has been established to assess the SA of the molecular system on the synthetic feasibility scale ranging from 1(lower) to 10 (higher). Lower values of SA support synthetic feasibility while higher values depict the difficulty in synthesis [181]. The SA score can be predicted in SwissADME, online database software that works on fragment utilities (Table 6).

**Table 6.** Available programs for the analysis of the molecular and pharmacokinetic parameters.

Server/software	Availability	Developer	City/country	Website link
Molinspiration	Free/open-source platform	Spin-off of Bratislava University	SlovenskyGrob/Slovak	https://www.molinspiration.com/
Molsoft	Free/open-source platform	Ruben Abagyan	CA/USA	http://molsoft.com/mprop/
SwissADME	Free/open-source platform	Swiss Institute of Bioinformatics	Lausanne/Switzerland	http://www.swissadme.ch/
OSIRIS	Free/open-source platform	Actelion/Idorsia Pharmaceuticals Ltd.	Allschwil/Switzerland	http://www.openmolecules.org/datawarrior/
Phase	Commercially available	Schrödinger, LLC	New York/USA	https://www.schrodinger.com/phase
DruLiTo	Free/open-source platform	Team Ankit Geete *et al.*	Punjab/India	http://www.niper.gov.in/pi_dev_tools/DruLiToWeb/DruLiTo_index.html
LigandScout	Commercially available	Inte:Ligand GmbH	Vienna/Austria	https://ligandscout.software.informer.com/
ACD Percepta	Commercially available	ACD/Labs	Toronto/Canada	https://www.acdlabs.com/products/percepta/index.php
Dragon	Commercially available	Talete	Milano/Italy	http://www.talete.mi.it/products/dragon_description.htm
E-Dragon	Free webserver	Institute of Bioorganic & Petroleum Chemistry	Kyiv/Ukraine	http://146.107.217.178/lab/edragon/start.html
Canvas	Commercially available	Schrödinger, LLC	New York/USA	https://www.schrodinger.com/canvas
RDKit	Free/open-source platform	GitHub and SourceForge	San Francisco (CA)/USA	https://www.rdkit.org/docs/source/rdkit.ML.Descriptors.MoleculeDescriptors.html

#### Ligand-based pharmacophore approaches

In ligand-based drug discovery evaluations, pharmacophore approaches have now proved their significance. The substructure of ligands that are necessary for the best possible bioactivity can be associated and categorized as a three-dimensional agreement of characteristics in three-dimensional space, which can be utilized for testing or employed in 3D-QSAR development [182–184]. Ligand-based pharmacophores are also produced by various online and offline tools. A set of data of varied ligands with recognized bioactivity versus the target is expected for pharmacophore development [185]. For ligand-based pharmacophore modeling, the applicable ligands should be interacting with a similar pocket and have the same binding interactions. The individual test set is used to validate the obtained pharmacophore [186]. It is then used for simulated exploration of a drug candidate from the libraries of unverified molecules, in which molecules are held as a possible lead. The utilization of molecular topographies instead of structural groups in describing vital activities is the key benefit of pharmacophore modelling, it has also permitted the evaluation of new drug candidates with distinct structures [187]. Likewise, for target describing and poly-pharmacological evaluation, pharmacophores can also be applied. This is specifically valuable when designing novel antibiotics or antiviral drugs to prevent. Due to the frequent similarities among pharmacophore as well as 3D-QSAR approach, these approaches have been applied in correlation for many LBDD [188,189].

#### Scaffold-based approaches

LBDD tools play a very significant role in CADD. Scaffold-based approaches are very common and reasonable, in which drug candidate molecules can be optimized and designed taking advantage of the chemical structural similarity with the known inhibitors or modulators [190]. Against a definite target protein, a representative system needs a reference chemical molecule along with currently available bioactivity information. This is formally selected as a model to find novel drug molecules from the available chemical database library and to select for further lab testing [191]. Scaffold-based molecular descriptors, as well as parameterizations, can be directly applied to describe a compound's efficiency based on similarities. one to three-dimensional structures were used by the descriptors for the further analysis, in which 1D descriptors include worldwide druglike parameters like MW, milogP, HBA, HBD, RB, and MR, etc., similarly 2D descriptors associated with the connectivity include topological parameter like TPSA, aromaticity, etc. and 3D descriptors linked with the geometrical parameters are volume, size, shape, and polarity, etc. [192,193] Fingerprints are utilized to describe a model and databank library by interpreting structural topographies like pharmacophoric elements, substituted/functional groups, topology/route, and circular/radial [193]. To link coordination information to finding basic structural topographies, deliberation arrangement is essential to ration and feature the significance of several characteristics of a drug candidate [194]. Tanimoto coefficient utilizes the ratio of common topographies among the respective fingerprint sets and is utilized generally for the assessment of variable vectors which lies between 1 and 0 [195]. Some other well-known structural measures are the Manhattan distance, Euclidean distance, Dice index, and Cosine coefficient [194]. There is not a common process that can be measured at the top of the complete series of identified target proteins as well as a library of molecules, each as every known procedure has its dataset effectiveness [194,195]. Therefore, both 2D and 3D structural approaches have been effective in finding a lead for diverse target proteins and have been recognized to have similar or improved upgrading than docking [196]. Although structure-based methods are recognized for their proficiency, there is a risk of gaining small range lead as maximum structural procedures are extremely reliant on the participating structures utilized to estimate descriptors [195].

##### ADMET properties in drug discovery

Evaluation of ADMET properties at a primary stage of drug development is needed for the preclinical analysis [197]. Computer-aided studies have advantages for the analysis of the ADMET properties, whereas experimental assessment is a time-consuming and expensive process (Table 7) [181].

**Table 7.** Available programs of the ADMET parameters analysis.

Server/software	Availability	Developer	City/country	Website link
admetSAR 2.0	Free webserver	East China University of Science and Technology	Shanghai/China	http://lmmd.ecust.edu.cn/admetsar2/
SwissADME	Free webserver	Swiss Institute of Bioinformatics	Lausanne/Switzerland	http://www.swissadme.ch/
ChemoSophia	Free webserver	Laboratory of Computational Modelling of Drugs, South Ural State University,	Chelyabinsk/Russia	http://www.chemosophia.com/soft.php
FAF-Drugs4	Free webserver	UMRS Paris Diderot-Inserm 973	Paris/France	https://fafdrugs4.rpbs.univ-paris-diderot.fr/
QikProp	Commercially available	Schrödinger, LLC	New York/USA	https://www.schrodinger.com/qikprop
PreADMET	Free webserver	Yonsei Engineering Research complex, Yonsei University	Seoul/Korea	https://preadmet.bmdrc.kr/adme/
ADMET Predictor	Commercially available	Simulations Plus	CA/USA	https://www.simulations-plus.com/software/admetpredictor/
ADMET	Commercially available	BIOVIA	San Diego (CA)/USA	https://www.3dsbiovia.com/products/collaborative-science/biovia-discovery-studio/qsar-admet-and-predictive-toxicology.html

###### AdmetSAR

AdmetSAR (ADMET structure-activity relationship) is a free online regularly updated database. AdmetSAR server frequently updates existing data related to ADMET properties from recent literature [198]. Furthermore, the admetSAR server provides access to 96,000 unique compounds covering more than 50 categories with ADMET-allied properties such as Caco-2 cell permeability, blood–brain barrier penetration (BBB), Ames's toxicity assessment, human intestinal absorption (HIA), oral bioavailability, and carcinogenicity, etc. which are useful for the analysis of the proposed drug compounds [199].

###### SwissADME

SwissADME is an openly available tool [200]. The key features of this software are systematic analysis, alternate input methods, and ease of analyzing multiple molecules. SwissADME is also included in the Swiss drug design workspace established by the molecular modeling group of the Swiss Institute of Bioinformatics (SIB). Cheminformaticians have also established many molecular descriptors based on chemical structures. The most known method is molecular fingerprinting (FP), which is made up of the sequence of bits explaining a biological activity, ADMET properties, and drug-likeness [181,201,202].

###### PreADMET

PreADMET is also an online database server for estimating the ADMET-related properties to assess the pharmacokinetics and druglikeness of the molecular library including ADME, druglikeness, molecular descriptor, and toxicity prediction [203]. PreADMET processes mol files for the evaluation of proposed parameters. Some of the common parameters are human intestinal absorption (HIA), plasma protein binding distribution, Caco-2 Cell permeability, blood–brain barrier penetrability (BBB), canine kidney cell permeability, skin permeability, and Madin-Darby. PreADMET also predicts the mutagenicity and carcinogenicity of the proposed compounds (Table 7).

####### Blood–Brain Barrier (BBB)

BBB is a significant ADMET property to assess whether the probable drug applicant can cross over into the brain or not. It aids in decreasing the unwanted effects and reactions as well as to ascertain the efficiency of the drug inside the brain [204,205].

####### Human Intestinal Absorption (HIA)

HIA is another essential factor of significant consideration during the drug design, optimization, and selection of orally active drug molecules. The intestine is the most important site of absorption for an orally consumable drug and an important parameter in the search for potential drug-likeness of a lead molecule [206,207].

####### Caco-2 permeability

Caco-2 is a human colon epithelial cancer cell line; it has a substantial role in drug development. Positive results represent the Caco-2 permeability. Mostly, it is applied for the analysis of drug absorption in the human intestine [208,209].

####### Ames test

Ames test is also known as the *Salmonella typhimurium* reverse mutation assay. Ames test is useful for the identification and analysis of genetic damage and frameshift mutations. It is well known that DNA is identical in all organisms. Ames test is executed with *Salmonella Typhimurium* and *Escherichia coli* to recognize the possible human carcinogens [210].

##### Toxicity Assessment

The development of drugs with minimum toxicity is the need of the hour. The analysis of the toxicity of any chemical entity is a very challenging task. In the last decade, the majority of discovered chemical entities were either ignored or found hazardous upon human exposure [211]. After suitable carcinogenicity or mutagenicity tests, these chemical entities can be subjected to human beings. Skin problems are also caused by considerable toxicity usually by exposure to several chemical entities [202]. Another most common effect of chemicals appears during pregnancy affecting the fetus and postnatal growth. For the prevention of these effects, the application of computer-aided studies has become very useful before the experimental stage (Table 8).

**Table 8.** Available programs/software of the toxicity parameters, metabolic transformation, and bioactivity score analysis.

Server/software	Availability	Developer	City/country	Database
OSIRIS	Free/open-source platform	Actelion/Idorsia Pharmaceuticals Ltd.	Allschwil/Switzerland	http://www.openmolecules.org/datawarrior/
PreADMET	Free webserver	Yonsei Engineering Research complex, Yonsei University	Seoul/Korea	https://preadmet.bmdrc.kr/toxicity/
CLC-Pred	Free webserver	Institute of Biomedical Chemistry and Pirogov Russian National Research Medical University	Moscow/Russia	http://www.way2drug.com/Cell-line/
admetSAR 2.0	Free webserver	East China University of Science and Technology	Shanghai/China	http://lmmd.ecust.edu.cn/admetsar2/
Lazar toxicity predictions	Free webserver	Albert-Ludwigs-Universität Freiburg	Freiburg/Germany	https://lazar.in-silico.ch/predict
Metatox	Free webserver	Institute of Biomedical Chemistry and Pirogov Russian National Research Medical University	Moscow/Russia	http://www.way2drug.com/Cell-line/
VirtualToxLab	Free webserver	University of Basel and Biographics Laboratory	Basel/Switzerland	http://www.biograf.ch/index.php?id=projects&subid=virtualtoxlab
ToxPredict	Free webserver	OpenTox	Zeiningen/Switzerland	https://opentox.net/library/toxicity-prediction
PASS Online	Free webserver or commercially available	Institute of Biomedical Chemistry and Pirogov Russian National Research Medical University	Moscow/Russia	http://pharmaexpert.ru/Passonline/index.php

##### Petra/Osiris/molinspiration Analyses

###### Petra calculations

PETRA online software (http://www2.chemie-unierlangen.de/services/) uses empirical methods for calculating the physicochemical characteristics of organic entity. The study team of Prof. J. Gasteiger has developed these methodologies over the past 20 years, and they are all empirical in nature [212]. The heats of formation, bond dissociation energies, sigma charge distribution, p-charge distribution, inductive effect, resonance effect, delocalization energies, and polarizability effect can all be measured in terms of chemistry [213].

###### Osiris Data Warrior

Osiris data warrior (https://openmolecules.org/datawarrior/) is a powerful data visualization and analysis program that delivers collaborative assessments for a pictorial representation of data. Data screens permit vigorous concentrating on data divisions [214]. Osiris Data warrior calculates the drug-likeness the structure is effective and predicts the outcomes in the form of color representation. Hazardous effects like mutagenicity as well as reduced intestinal absorption are represented by a red color code, while a green color shows drug definite action [215]. The software draws the structure of chemical entities and computes several drug-relevant properties if the structure is effective. The calculation is established with a pre-computed set of structural fragments that contributes to the rise in toxicity warnings.

###### Molinspiration calculations

The Molinspiration (http://www.molinspiration.com/services/) software is used to calculate cLogP (octanol/water partition coefficient) as the total of fragment-based contributions and correction factors [216]. The procedure is exceedingly reliable and can handle almost all organic and organometallic compounds. Based on the methods described by Ertl *et al.*, the molecular polar surface area (TPSA) is determined as the sum of fragment contributions. Also investigate polar fragments with O- and N-centers. Drug absorption, including intestinal absorption, bioavailability, Caco-2 permeability, and blood-brain barrier penetration, has been demonstrated to be extremely well described by PSA [212,213].

#### Metabolic transformation

Metabolizing enzymes create some problems during the absorption of an offered drug molecule so metabolic stability is a key feature in drug optimization [217]. Evaluation of the metabolic sites is an essential feature to assess metabolic stability and prediction of possible pharmacological activity [218]. In living organisms, drug metabolism typically occurs in the liver and gut epithelia. Experimental analysis of metabolic sites in the proposed molecule may require a large amount of time and expertise. Computer-aided metabolism predictions display appropriate results, using a simple protocol [219].

##### Metabolic transformation prediction

**Figure 11.**
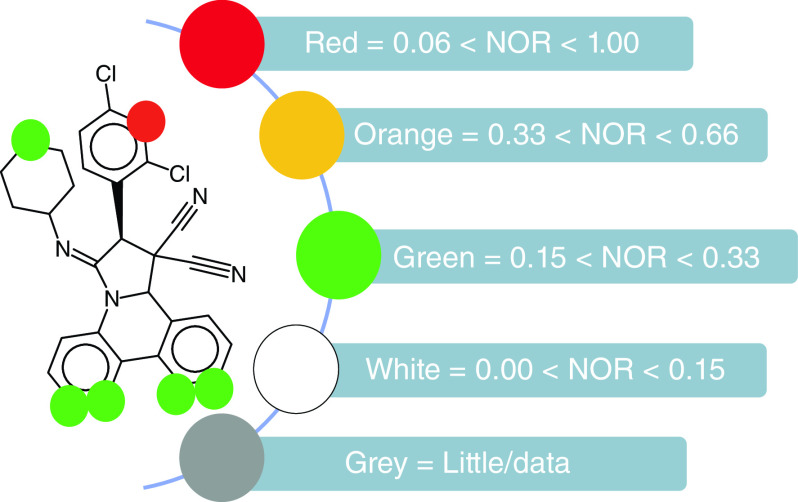
MetaPrint2D window. NOR: Normalized occurrence ratio.

The metabolic site of the proposed molecules can be calculated with the help of MetaPrint2D. MetaPrint2D analyzes drug metabolism by comparing the chemical compound in question with the available metabolic transformation data described by the researchers [181]. MetaPrint2D, now a part of Bioclipes v2.6.2 requires a MOL/SDF format as an input file and predicts the results as color presentations [220]. Different colors represent the normalized occurrence ratio (NOR). A high value of NOR depicts a frequently described metabolic site in the available databank [215,220,221]. The virtual probability of metabolism at a specific point is successfully calculated by MetaPrint2D. The color indications associated with the NOR metabolic sites are shown in Figure 11.

#### Bioactivity score

Quality drug designs are stabilized with the help of molecular properties and structural features for instance hydrophobicity, electronic distribution, hydrogen bonding ability, molecular magnitude, and elasticity which stimulate the performance of a molecule in an existing entity as well as bioavailability, transport, and likeness to proteins, etc. In the process of exploration of a possible drug candidate, it is essential to recognize the binding sites on specific proteins [222,223].

##### Molinspiration for the calculation of bioactivity score

Molinspiration is an online-based free server for the analysis of bioactivity scores along pharmacokinetic properties, usually significant drug targets like GPCR ligand, ICM, NRL, KI, PI, and EI. It has a broad range of cheminformatics tools associated with molecular operation and requires simple handling by SMILES/SDF file [201]. Stabilization of molecules, generation of the tautomer, fragmentation of molecules, and designing of several molecular parameters required in QSAR, molecular modeling, and drug design can also be performed with the help of the software [224]. Molinspiration does molecular calculation with the help of the existing information in the molecular database and controls fragment-based virtual screening. It quickly calculates the biological activity scores based on standard models stored in the Molinspiration in-house library consisting of thousands of activated compounds. Calculations can be completed in a single run against all targets [214,215].

##### Drug targets

A drug target can be a receptor protein which is usually an essential protein in the natural system whose action can be transformed by binding a drug molecule expected to have a necessary therapeutic conclusion. Some most acceptable drug targets are -

- G protein-coupled receptors (GPCR) (target more than 50% of drugs)
- Enzymes (mainly protein kinases, proteases, esterases, and phosphatases)
- Ligand-gated ion channel
- Ion channels Modulators

###### G protein-coupled receptor (GPCR)

GPCR is generally mentioned as the seventh *trans*-membrane receptor. It includes an enormous protein family of receptors that identify molecules when they penetrate the cell and stimulate signal transduction paths within the cell's primary cellular response [225,226].

###### Protein kinase (PK)

Protein kinases are a part of the kinase enzyme family. The enzyme is utilized by transforming other proteins through the chemical addition of the phosphate group (phosphorylation). Approximately 30% of the total human proteins are improved by the kinase action. Numerous diseases mostly cancer can be a cause of disturbance in the kinase activity (kinases regulate cell growth, movement, and death) [227].

###### Ion channel modulators

Ion channel modulators (ICM) permits the movement of charge ions via the cell membrane. They are included in an array of biological pathways of several muscle cells. They replenish the equilibrium of ions across a membrane. There are two main ion channels: ligand-gated ion channels (LGIC) as well as voltage-gated ion channels (VGIC) [215]. VGIC works on the basis of the variance in the voltage that arises due to the variation in voltage across the cell membrane, permitting the ions to transfer across the membrane. The binding ability of a small molecule toward the channel depends on the LGIC and changes the channel to allow a moment of ions by developing the charge on the channel protein [162].

###### Nuclear receptor ligand

Nuclear receptors are responsible for the identification of steroids, proteins, and other molecules. These receptors work with other proteins in conjugation, in the expression of specific genes, in that way regulatory homeostasis and metabolism. They also can bind to DNA and be categorized as transcription factors. A ligand interaction with the nuclear receptor allows the conformational changes in the receptor and stimulates the receptor to regulate gene expression [228].

###### Enzyme inhibitors

Enzyme inhibitors (EI) act in conjugation with the enzymes to inhibit their functioning. Numerous drugs work as enzyme inhibitors by blocking the activity of enzyme [229,230]. Table 8 summarizes some software to produce important drug parameters.

### Quantitative structure–activity relationship-based approaches

The quantitative structure–activity relationship (QSAR) is a structure-based method, which is useful for relative evaluation, a process that is applied to examine the association between the structural and physicochemical parameters of a drug candidate with different biological activities [231]. The desired activity of drug candidates is estimated through the QSAR studies based on the evidence that ligand two/three-dimensional assets can deliver evidence to create an arithmetical model of the anticipated biological activity [232]. The arithmetical model is produced on a suitable information set, involving structures by recognized bioactivity alongside a definite receptor, which essentially be the monitor as well as pre-treated. The conformational selection as well as alignment includes the advanced dimensions of QSAR modelling, which permits the recognition of functional groups that are involved in the activity [233]. In this instance, it is significant to observe the minimum energy conformation is not continually comparable to the bioactive conformation [234] and that the drug candidate selected for the analyzing set should relate to the identical interaction site [235]. Pharmacophore development for drug candidate alignment is more suitable as it associates molecules based on the characteristic relationship instead of the chemical substructure. Molecular descriptors employed in the template should be carefully selected to prevent auto-association [236]. Recently QSAR-based algorithms have incorporated CoMFA (Comparative Molecular Field Analyses) and CoMSIA (Comparative Molecular Similarity Indices Analysis), together [237–239].

### Density functional theory (DFT)

In the 1960s quantum mechanical approach established DFT, which has wide applications such as in physics, physical chemistry, computer-aided chemistry, and material science, to evaluate the electronic properties of an atom/molecular system [240,241]. Two Hohenberg-Kohn (HK) theorems are utilized in DFT. First, three spatially defined electron intensities are employed to describe the ground state of the atomic/molecular system. Second, the exact ground-state electron intensity can be optimized by the HK theorem defines as energy-efficient [242,243]. The utilization of DFT evades the computer-aided expenditure of conventional approaches including the Hartree-Fock (HF) theory, meanwhile, DFT depends on the principle that energies, complex motions as well as lone-pair links can be obtained from the electron prospect density, as an alternative to exploitation of wave-functions. For a molecular system, the Schrödinger wave equation can be resolved. It requires broad computer-aided parameters and is difficult to solve for a multiple-body system. Therefore, DFT is a very widespread tool in numerous computer-aided areas because it is utilized as a comparable and efficient substitute for the Schrödinger equation [244]. In the research of novel drug candidates, DFT has been observed to be applicable for evaluation, catalytic action, SAR studies as well as inhibiting effectiveness. Some researchers employed DFT to boost their preliminary studies involving a shift in inhibitor interaction mode in the target protein. Even with the realization and recognition of DFT, it nonetheless consumes inadequacies due to the involvement of extensive calculations.

Systems primarily encompassing dispersing forces like gaseous as well as biomolecular systems are difficult to describe through DFT [244]. Nevertheless, numerous evaluations consume previously explored the presence of dispersal forces to expand this approach. Additionally, the most important restraints of DFT utilization in computer-aided chemistry involve the categorization of comprehensive possible energy surfaces, charge-transfer excitations, as well as transition states [245–247].

### Combined approaches

Currently, along with the range of openly available tools as well as structural data for DDD, it is ordinary to discover findings that utilize a sequence correlation of structure- as well as ligand-based methodologies instead of limited use of each [248]. Moreover, a combination of these approaches frequently delivers improved findings [249]. Likewise, the positive features of a technique can overcome the constraints of the other, in an extremely balancing DDD route [250]. Combined *in silico* systems consist of successive and comparable approaches, however, hybrid approaches have also previously been built. Progressive approaches include the straight employ of computer-aided approaches with the target of improving the discernment of the VS system by constantly decreasing the figure of possible lead instead of experimental estimation [251]. Still, it has been proven that ligand- as well as structure-based approaches have comparatively improved and regularly produced lead molecules, demonstrating that these approaches are superior employed in equivalence instead of in sequence [252,253]. Combined applications, via immediate engagement of different computer-aided tools, frequently delivers a more distinct lead profile [254]. On the other hand, outcomes from these approaches are regularly combined to generate a final rating, and many computer-aided leads are found from this methodology [255,256].

## Drug repurposing

**Figure 12.**
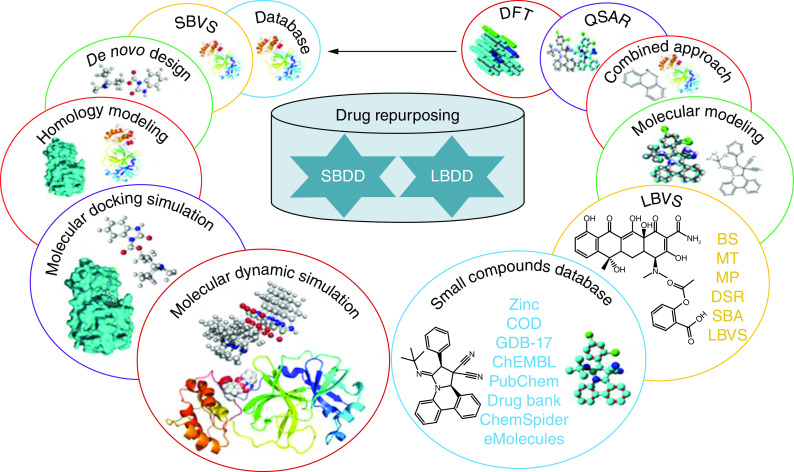
Effective computer-aided drug design pipeline.

Enhancement in the productivity of the pharmaceutical research is a big challenge to deal with [257]. To fill the productivity gap, drug repurposing has emerged as a potential solution as the technique aims at exploring the unknown facets of an already marketed drug to cure different disease conditions hence involving a low cost and time [258]. As compared with the traditional protocol of drug discovery, the method is highly advantageous as it was focused on already tested and known drugs that have been proven benign in humans and the risk of clinical failure is minimal [259]. These benefits have evoked the pharmaceutical industry, medical chemistry labs, and government to further explore the method to come up with quick yet systematic medicinal solutions. As it is well established now, that no drug so far has been developed to cure COVID-19 and hence drug repurposing has emerged as a viable strategy to discover a potential drug candidate from a vast pool of marketed drug moieties to combat SARS-CoV-2. Broad-spectrum antiviral agents (BSAAs) have been touted as promising drug repurposing candidates [259]. The strategy is based on the mechanism of replication of the virus and host interactions which may be similar in two or more viral families [260]. A few known antiviral agents have undergone Phase II trials. Lopinavir/Ritonavir is a drug mixture directing viral protease and is approved for the treatment of HIV and influenza. Umifenovira membrane synthesis inhibitor directing viral transfer. These drugs are now being considered in diverse transformations in Phase IV of a clinical test for pneumonia-related through COVID-19 [261,262]. However, drug repurposing is a complicated process and needs a systematic approach to identify the potential of drugs. Various computational approaches have been developed for accurate predictions including inverse docking which docks a current drug in the probable interaction pockets of disease targets (Figure 12) [263].

Therefore, drug repurposing is a process of discovering new therapeutic uses for existing drugs, by exploring their potential benefits beyond their originally intended purpose. It involves evaluating drugs already approved by regulatory agencies such as the FDA to develop new uses, which can reduce the development time and costs associated with bringing new drugs to market. This approach offers opportunities to identify promising drug candidates that can be repurposed with the potential to improve the lives of patients [264–266]. Drug repurposing, also known as drug repositioning, is the process of discovering new therapeutic uses for existing drugs that are already approved for other indications [264]. This approach presents several advantages, benefits, and applications in drug discovery and development.

### Advantages

■ Reduced costs and time: since repurposing studies leverage existing knowledge of drugs, they are less expensive and time-consuming than *de novo* drug discovery programmes, which require extensive testing and screening of large libraries of compounds.
■ Lower failure rates: repurposing studies rely on drugs that have already been approved for other indications, and their safety and toxicity profiles are well known, resulting in lower failure rates and faster progress through clinical trials.
■ Improved patient outcomes: repurposing can lead to the discovery of new treatments for currently unaddressed or poorly treated diseases. This approach can potentially lead to improved patient outcomes and lower morbidity and mortality rates.

### Benefits

■ Improving public health: repurposing can result in the development of new therapies for diseases with limited treatment options, such as rare diseases or neglected diseases, improving public health outcomes.
■ Sustainable development of treatments: repurposing can reduce the need for *de novo* drug discovery, which often relies on chemical synthesis, which generates large amounts of waste materials that can negatively impact the environment.
■ New market opportunities: repurposing can lead to the expansion of drug markets into new therapeutic areas, increasing the efficacy of existing drugs, and providing new opportunities for drug manufacturers.

### Applications

■ Finding new uses and indications: repurposing can identify new clinical uses for drugs that have already been approved for other indications, making them promising drug candidates for diseases with unmet medical needs.
■ Improving safety and efficacy: repurposing can be used to enhance the safety and efficacy of existing drugs by identifying new indications, dosing regimens, and drug combinations to improve the effectiveness of the drug.
■ Developing personalized treatments: repurposing can be used to develop personalized treatments for individual patients, based on their genetics, health history, and biomarkers, by identifying new uses for existing drugs that match the needs of specific patients.

In conclusion, drug repurposing presents several advantages, benefits, and applications in drug discovery and development. This approach can lead to the discovery of new uses for existing drugs, improve the safety and efficacy of treatments, and increase the sustainability of drug development programs. Hence, it is an important strategy for the pharmaceutical industry and researchers to consider. Here are few best examples of drugs that were discovered using drug repurposing:

■ Viagra: originally developed as a treatment for high blood pressure, was repurposed as a treatment for erectile dysfunction [265].
■ Thalidomide: originally developed as a sedative and anti-nausea medication for pregnant women, was repurposed as a treatment for multiple myeloma [266].
■ Metformin:, originally developed as an anti-diabetic medication, has been repurposed for the treatment of cancer [267].
■ Amantadine: originally developed as an anti-viral medication, has been repurposed for the treatment of Parkinson's disease and chronic fatigue syndrome [268].
■ Thioridazine: originally developed as an antipsychotic medication, has been repurposed for the treatment of antibiotic-resistant bacteria [269].
■ Aspirin: originally used as a pain reliever and anti-inflammatory medication, has been repurposed for the prevention of heart attacks and strokes [270].
■ Fluoxetine: originally developed as an anti-depressant medication, has been repurposed for the treatment of premenstrual dysphoric disorder and bulimia nervosa [271].
■ Doxycycline: originally developed as an antibiotic medication, has been repurposed for the treatment of rheumatoid arthritis and periodontitis [272].
■ Gabapentin: originally developed as an anti-epileptic medication, has been repurposed for the treatment of neuropathic pain and migraines [273].

### Tools & aids for effective repurposing

#### Auto in-silico consensus inverse docking

The auto *in*-*silico* consensus inverse docking (ACID) (http://chemyang.ccnu.edu.cn/ccb/server/ACID) approach works taking into consideration of inverse docking as an effective repurposing principle. Inverse docking works on the ‘one ligand many targets’ approaches, which was initially performed against small molecules binding with many macromolecular targets. The top drug targets were touted as the potential drug repurposing sites. Tools like INVDOCK, TarFisDock, PDTD, and id Target have been used for the repurposing [274]. Permutations and combinations involving different docking algorithms can lead to a higher success rate [275]. Elaborate web-based software, ACID with a simple handling protocol has been intended for flexible calculation consisting of the followings: (i) an automatic trustable docking workflow tool, (ii) more than 2086 approved drugs are found in databank (iii) From 30 therapeutic areas, total 831 receptor structures from PDB format present in the database (Table 9).

**Table 9.** Server/software of the drug repurposing.

Database/software	Description	Developer	City/country	Web link
ACID	A free tool for drug repurposing using consensus inverse docking strategy. Auto *in silico* Consensus Inverse Docking (ACID) is used for drug repurposing based on the consensus inverse docking method, designed to evaluate the binding affinities between each protein and small molecules.	The Yang Group	Wuhan, Hubei/China	http://chemyang.ccnu.edu.cn/ccb/server/ACID
ADReCS-Target	Adverse Drug Reaction Classification System-Target Profile (ADReCS-Target) provides comprehensive information for illustrating ADRs caused by drug interactions with protein, gene and genetic variation.	State Key Laboratory of Stress Cell Biology	Xiamen,Fujian/China.	http://bioinf.xmu.edu.cn/ADReCS-Target/
BindingDB	Small molecules annotated with bioactivity data.	Skaggs School of Pharmacy & Pharmaceutical Sciences	San Diego(CA)/USA	http://www.bindingdb.org/bind/index.jsp
ChemProt	A resource of chemical-protein interactions. The server is a compilation of over 1,100,000 unique chemicals with biological activity of more than 15,000 proteins.	Center for Biological Sequence Analysis	Lyngby/Denmark	http://www.cbs.dtu.dk/services/ChemProt/ChemProt-2.0/

#### Adverse drug reaction classification system (ADReCS) -target profile

ADReCS offers extensive data on ligand-receptor interaction and this server also contains up to 66,573 per pair interactions, out of which 1710 are proteins, 2613 are genetic variations, and 63,298 are gene and advanced drug reaction (ADR) interactions. This server is very beneficial for the online evaluations of HTS of a drug candidate before clinical testing. It is also very helpful to distinguish drug molecules along with superior ADR activity [276].

#### BindingDB

BindingDB (www.bindingdb.org), is a freely available databank containing 1,439,799 experimental binding affinity values of receptors and druglike small molecules, offering easy and advanced finding options [277].

#### ChemProt

ChemProt2.0 provides a pharmacological map connecting the bioactivities of compounds and proteins based on more than seven million interactions collected from several databases that interpret compounds, proteins, and diseases [278].

A good computational drug repurposing should facilitate new avenues in terms of the exploration of new drug compounds and diseases. The connectivity between the drug and disease depends upon the drug or disease-induced gene expression profile (GEPs). Molecular interactions, side effects, or disease indications are also important parameters to explore before drug repurposing [279].

## Conclusion

Design, development, and production of a novel safe, and effective drug candidate with a significant therapeutic prospect are currently a major challenge to arise for researchers. Drug discovery is a long process and required a large amount of money. Many factors influence the efficiency of the drug candidate such as molecular weight, bioavailability, toxicity profile, and an excess of other dynamic pharmacokinetics and pharmacological limitations. CADD is a proficient method in the field of DDD, the origin of CADD helps and easily makes the estimation and structural depiction of novel hits and has developed a useful and descriptive approach. CADD can lead the tactic to a molecular behavior. The CADD technique has boosted progress rising by the capable approximation of drug-likeness in a limited period. A wide range of molecular descriptors, pharmacokinetics, and pharmacodynamics, ADME, and toxicity parameters can be calculated with the help of computer-aided programs. Here, numerous open software such as Molinspiration, Osiris Data warrior, admetSAR, SwissADME, PreADMET, MetaPrint2D, etc., and the parameters such as molecular weight, clogP, hydrogen bond donor, hydrogen bond acceptor, molecular refractivity, rotatable bond, etc. estimated and discuss in detail. The bioactivity score of the drug candidates can be predicted for GPCR ligand, ICM, NRL, KI, PI, and EI calculated with the help of Molinspiration. ADMET parameters are easily predicted by using the admetSAR, PreADMET, and SwissADME databases. Like ADMET constraints, understanding the metabolism of a novel lead molecule is also dynamic which is presence predicted by MetaPrint2D tools. MetaPrint2D are highlighting the positions of molecular structure and provide information on how it is effective to be metabolized, it is really for the formation of the drug candidate. The assessments of toxicity and drug-likeness are also major factors of any drug candidate and can be predicted by Osiris Data Warrior. Molecular docking, as well as molecular dynamics, has come to be a vital approach for CADD, their combined performance displays a key role in pharmacology, medicine, biology, environmental science, agronomy, etc. Their expansion is influenced by the improvement and saturation of many interdisciplinary subjects. All at once, the quick progress of different interdisciplinary subjects has moreover produced advantageous environments for the improvement of CADD.

The molecular docking and molecular dynamics approaches offer features for calculating the nature and interaction profile of drug-target complexes and deliver valuable orientation and theoretic provision for promoting experimental research. Both methods have come to be the most acute and extensively used approaches in exposing biological as well as molecular mechanisms, exclusively in the calculation of complex structures at molecular or atomic stages. Currently, biological data are extensively increasing; the prominence and complexity of molecular docking and molecular dynamics tools are progressively established. The new complex biological problems are further arising due to these the contribution and applications of both tools are more desirable. At this time, when predictable models are potential the microsecond scale, conformational alterations, or ligand interaction can be successfully simulated. The enlargement of the virtual tools, particularly the usage of GPUs and the advances prepared in the optimization of MD procedures such as coarse-grained, permits us to transfer from the investigation of single structures of the source of the molecular modeling and confessional groups. Conformational groups are a considerably improved depiction of actual biological macromolecules and the description for flexibility and dynamic assets, its simplicity, and the findings are equal with experimental consequences. To distinguish the interaction profile of ligand-target complex is a vigorous step in search of the lead, molecular docking, and molecular dynamics are an outstanding approach for CADD prominent to the prediction of the interaction profile of ligand-target complex. Here, some open docking tools are discussed such as AutoDock, AutoDock Vina, AutoDock FR, and iGEMDOCK. These computer-aided approaches are suitable and come to be the core tools of present CADD research, through it we can discover the auspicious drug candidate within a short period and low cost. CADD approaches always offer an expectation for advancement in drug discovery research.

## Future perspective

As the field of computer-aided drug design and discovery continues to grow, it is expected that new technologies and algorithms will be developed over the next 5–10 years. The use of AI and machine learning will become increasingly important in identifying potential drug candidates and predicting their efficacy, toxicity, and pharmacokinetics. One of the major challenges that researchers face is the ability to accurately predict the binding affinity of a candidate drug molecule to its target protein. While computational methods have improved significantly in recent years, further research is needed to develop more accurate models and algorithms for predicting binding affinities. This will require the use of larger data sets and the incorporation of more complex molecular interactions. Another challenge is the ability to predict the behavior of drug molecules *in vivo*. While *in silico* models can be used to predict the pharmacokinetics and toxicity, there is a need for more accurate and comprehensive models that consider the complex interactions between the drug molecule, different target, and the biological system. To address these challenges, researchers need to develop new algorithms and computational tools that can effectively integrate molecular modelling, machine learning, and big data analytics. In addition, there is a need for collaborations between different disciplines, such as computer science, chemistry, biology, and medicine, to achieve a truly comprehensive understanding of drug design and discovery. Overall, the field of computer-aided drug design and discovery is expected to continue to have a significant impact on the pharmaceutical industry and on society. Continued research and development will be needed to take full advantage of the opportunities presented by these technologies and to address the challenges inherent in drug discovery and development.

**Executive summary**

**Structure-based drug design tools**

- Structure-based drug design (SBDD) tools assist in the development and optimization of new drugs.
- They rely on the 3D structure of a target molecule, often a protein, to identify potential drug candidates.
- In SBVS, computer algorithms screen large databases of compounds and identify those which bind to the target.
- These tools use molecular docking, and virtual screening, to predict the interaction between a drug candidate and a target molecule.
- SBDD tools help in designing drugs that are more specific, effective, and have fewer side effects.

**Structure-based virtual screening**

- Structure-based virtual screening (SBVS) uses the 3D structure of target proteins to identify potential ligand as drug candidate. It involves two steps: molecular docking and scoring.
- Docking involves computationally positioning small molecule compounds into the binding site of the protein.
- Scoring evaluates the binding affinity and stability between the ligand and the target. The results are used to prioritize compounds for further analysis and testing.
- This method allows for the screening of large compound libraries.
- Accuracy of the protein structure and limitations of the docking algorithms can affect the success of the screening.
- Fragment-based methods can be used in combination with SBVS to improve results. This approach has been successful in identifying potential drug candidates for a range of diseases, including cancer and infectious diseases.

**Ligand-based drug discovery tools**

- LBDS identifies new drug candidates by analyzing the interactions between a target and potential ligands. It relies on the knowledge of the three-dimensional structure of the target and its binding site, and the chemical properties of the ligand.
- LBDS can be used to design selective drugs with fewer side effects. It includes pharmacophore modeling, QSAR analysis, and molecular docking.
- Pharmacophore modeling involves identifying the chemical features of ligands that are necessary for binding to the target.
- QSAR analysis involves developing mathematical models relating the chemical structure of a ligand to its activity and using these models to predict the activity of new ligands.
- The success of LBDS depends on the accuracy of the computational techniques used.

**Drug repurposing**

- Drug repurposing refers identifies new uses of the existing drugs that were originally developed for a different purpose. Repurposed drugs may also be faster and easier to gain regulatory approval as their safety data is already established.
- Drug repurposing can involve either testing existing drugs on new indications or identifying new targets for existing drugs that are already known.
- The process of drug repurposing involves the use of computational approaches and HTS to identify potential drug candidates.

**Future perspective**

- The use of AI and machine learning will become increasingly important in identifying potential drug candidates and predicting their efficacy, toxicity, and pharmacokinetics.
- There is a need for collaborations between different disciplines, such as computer science, chemistry, biology, and medicine, to achieve a truly comprehensive understanding of drug design and discovery.
